# A molecular toggle after exocytosis sequesters the presynaptic syntaxin1a molecules involved in prior vesicle fusion

**DOI:** 10.1038/ncomms6774

**Published:** 2014-12-17

**Authors:** Deirdre M. Kavanagh, Annya M. Smyth, Kirsty J. Martin, Alison Dun, Euan R. Brown, Sarah Gordon, Karen J. Smillie, Luke H. Chamberlain, Rhodri S. Wilson, Lei Yang, Weiping Lu, Michael A. Cousin, Colin Rickman, Rory R. Duncan

**Affiliations:** 1 Institute of Biological Chemistry, Biophysics and Bioengineering, Heriot Watt University, Edinburgh EH14 4AS, UK; 2 Edinburgh Super-Resolution Imaging Consortium, www.esric.org; 3 Centre for Integrative Physiology, University of Edinburgh, George Square, Edinburgh EH8 9XD, UK; 4 Strathclyde Institute of Pharmacy and Biomedical Sciences, 161 Cathedral Street, Glasgow G4 0RE, UK

## Abstract

Neuronal synapses are among the most scrutinized of cellular systems, serving as a model for all membrane trafficking studies. Despite this, synaptic biology has proven difficult to interrogate directly *in situ* due to the small size and dynamic nature of central synapses and the molecules within them. Here we determine the spatial and temporal interaction status of presynaptic proteins, imaging large cohorts of single molecules inside active synapses. Measuring rapid interaction dynamics during synaptic depolarization identified the small number of syntaxin1a and munc18-1 protein molecules required to support synaptic vesicle exocytosis. After vesicle fusion and subsequent SNARE complex disassembly, a prompt switch in syntaxin1a and munc18-1-binding mode, regulated by charge alteration on the syntaxin1a N-terminal, sequesters monomeric syntaxin1a from other disassembled fusion complex components, preventing ectopic SNARE complex formation, readying the synapse for subsequent rounds of neurotransmission.

## Abstract (web summary)

Synaptic vesicle fusion involves a multi-protein assembly called the SNARE complex that is tightly regulated both spatially and temporally. Here Kavanagh *et al*. show that after vesicle fusion and SNARE complex disassembly in the synapse, the SNARE protein syntaxin1a is sequestered in a monomeric form by munc18-1, preventing ectopic SNARE complex assembly.

## Graphical abstract

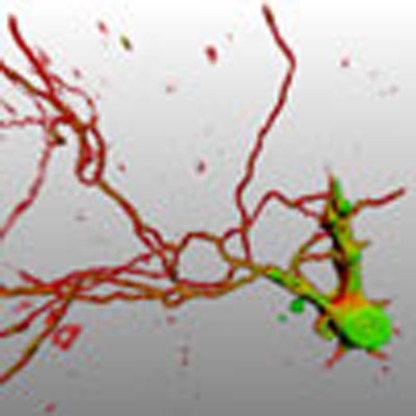

All forms of membrane fusion rely on a core family of SNARE proteins^1^. The synchronized action of a number of accessory proteins is also required to oversee the highly ordered and localized nature of SNARE mediated exocytosis (for review, see ref. 2). Sec1/Munc18 proteins (SM proteins) are a class of such accessory factors that are present at all SNARE-catalyzed membrane fusion sites^3^. It is known that munc18-01 and syntaxin1 (the principal SM protein and syntaxin involved in synaptic exocytosis) interact via at least two distinct modes; one with monomeric ‘closed’ syntaxin1a and the other involving its highly conserved amino-terminal (N-terminal) peptide motif^4^,^5^,^6^. Current hypotheses incorporate these data into models where munc18-01 and syntaxin1a interact using distinct binding modes depending on intracellular location and function^3^,^7^. However, whether munc18-01 interacts with syntaxin1a and functions in the late stages of synaptic vesicle fusion^7^,^8^,^9^ or whether it completely dissociates from syntaxin1a, or syntaxin1a-containing complexes, during exocytosis is still unspecified. Therefore, despite a large amount of biochemical, electrophysiological and ultra-structural data, the spatiotemporal arrangement of munc18-01 and syntaxin1a at a molecular level in living neuronal cells and particularly in central synapses remains undefined. Questions surrounding the molecular interaction starting point of the synaptic vesicle cycle have proven difficult to probe, principally because of a dearth of single-molecule resolution approaches. Here we employed imaging and spectroscopic approaches to quantify the distributions, movements and interactions of munc18-01 and syntaxin1a molecules in central synapses to identify directly the small number of molecules specifically involved with synaptic vesicle exocytosis and to identify the interaction pathway in synapses before, during and after synaptic vesicle exocytosis (Fig. 1a).

## Results

### Munc18-1 and syntaxin1a single-molecule dynamics

**Figure 1:**
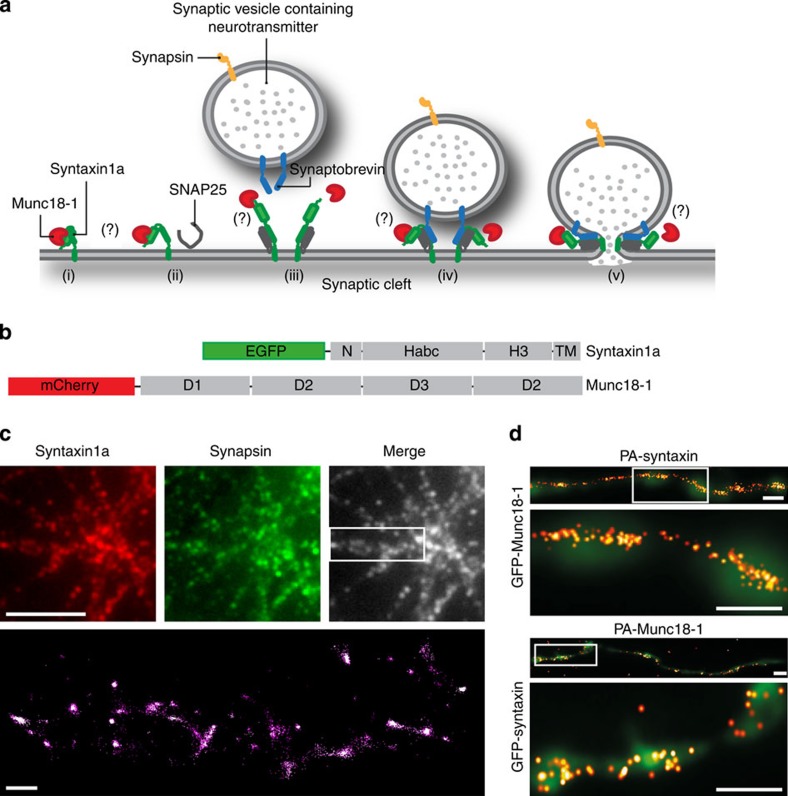
Munc18-1 and syntaxin1a single-molecule distribution in neurons. (**a**) Model of the proposed munc18-1 (red) and syntaxin1a (green) interactions at a synapse. Munc18-1 binds syntaxin1a in closed confirmation, preventing syntaxin1a from entering the SNARE complex and inhibiting membrane fusion (i) The binding mode of munc18-1 bound to syntaxin1a switches from the closed to open mode, allowing the formation of the binary t-SNARE complex (ii) SNARE binary complex with t-SNARE partner SNAP-25 in grey (iii). Ternary complex of open syntaxin1a, SNAP-25 and synaptobrevin required for membrane fusion (iv). Question marks represent uncertain points of syntaxin-munc18-1 molecular interaction in the synaptic vesicle cycle. (**b**) Schematic illustrating the syntaxin1a and munc18-1 constructs used in this study. (**c**) dSTORM map of immunodetected syntaxin1a (Alexa-647, upper left) and synapsin-EGFP (upper right) in cortical neurons. A merged image (grey, upper right) shows overlap. Lower panel: a dSTORM molecular map from the boxed area in the merge image shows the locations of single immunodetected syntaxin1a molecules concentrated in synapsin-positive synapses with sparse distribution elsewhere in the neuron. (**d**) PALM localization maps show single molecules of PA-mCherry-syntaxin1a or PA-mCherry-munc18-1 co-clustering with either EGFP-munc18-1 or EGFP-syntaxin1a, respectively. The boxed regions are displayed at a higher zoom (top panels). Scale bars, 500 nm. The distribution of heterologous munc18-1 and syntaxin1a fluorescent fusion protein molecules is similar to the endogenous pattern.

We previously developed fluorescent syntaxin1a and munc18-1 probes (Fig. 1b) that we showed target appropriately in neuroendocrine cells^5^,^10^,^11^,^12^,^13^,^14^, as well as being functional^5^,^10^,^14^ (also shown by others using similar constructs)^15^,^16^,^17^, engaging in protein–protein interactions in a predictable way^5^,^10^,^16^,^17^. Importantly, the single-molecule distributions, localizations and interactions of these essential presynaptic proteins have never been elucidated *in situ—*that is, in central neurons. We believe that quantifying protein–protein interactions at the molecular level, as opposed to the more common colocalization or bulk dynamic studies, is essential to allow progress to be made in our understanding of not just synaptic biology but of the gamut of cell biological questions. To probe and compare the molecular organization of endogenous and heterologous munc18-1 and syntaxin1a on the nanoscale, we employed both direct stochastic optical reconstruction microscopy microscopy (dSTORM^18^,^19^) and photoactivatable (PA) localization microscopy (PALM^20^,^21^). dSTORM involved the immunodetection of endogenous proteins with a fluorophore-conjugated antibody (Alexa-647) driven into a long-lived ‘dark-state’ using high-intensity laser illumination in the presence of a reducing buffer^19^. Single Alexa-647 fluorophores, conjugated to secondary antibodies, spontaneously re-emerge from this dark state, permitting the localization of individual epitopes separated in a time stack. Munc18-1 and syntaxin1a molecules were thus detected in chemically fixed cortical neurons (fixation was for 90 min to ensure complete immobilization). Subsequent co-staining against synapsin was performed to delineate presynaptic areas. dSTORM imaging revealed that both endogenous munc18-1 and syntaxin1a molecules (with the caveat that we detect immunolabelled complexes larger than the targets) accumulate at nerve terminals (Fig. 1c and Supplementary Fig. 1).

Next we used PALM, comparing the distribution of heterologous syntaxin1a and munc18-1 with the spatial pattern of endogenous molecules ascertained using dSTORM. To achieve the maximum resolution, we first examined chemically fixed cells co-expressing either PA-mCherry-Munc18-1/enhanced green fluorescent protein (EGFP)-syntaxin1a (EGFP provided diffraction-limited resolution data) or conversely, PA-mCherry-syntaxin1a/EGFP-Munc18-1. Positional information describing PA-mCherry-munc18-1 and PA-mCherry-syntaxin1a molecule localization was rendered into maps (Fig. 1d), where munc18-1 and syntaxin1a were seen to co-cluster with one another in varicosities (Fig. 1d). To determine whether these areas represented nerve terminals, neurons were transfected with PA-mCherry-syntaxin1a, fixed and co-immunolabelled against synapsin as before. PALM imaging confirmed that these varicosities represented synapses; single syntaxin1a (and so also munc18-1) molecules clustered at synapsin-positive synapses (Supplementary Fig. 1). By observing and measuring directly the molecular distributions of these endogenous and heterologous proteins in neurons for the first time, we reveal a molecular distribution, with sparse, individual molecules in processes but denser accumulations in varicosities, suggesting that syntaxin1a and munc18-1 are trafficked along axons before accumulation in presynaptic areas^22^,^23^.

**Figure 2:**
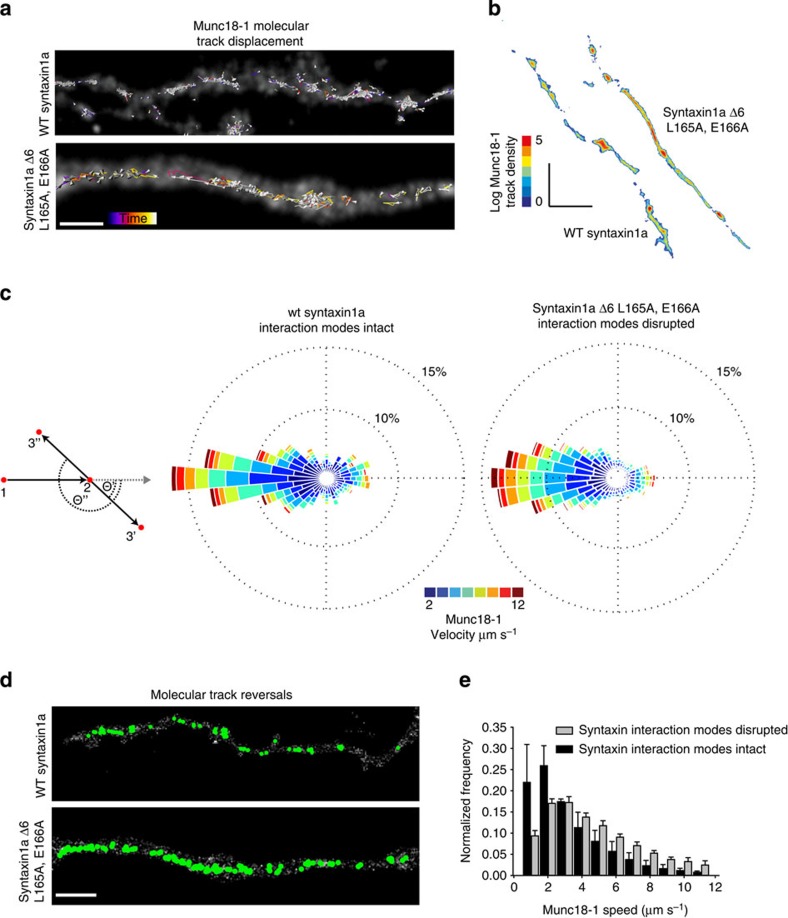
Munc18-1 molecular distributions, speeds and dynamics are syntaxin1a interaction dependent. (**a**) The as-the-crow-flies displacements of munc18-1 single-molecule tracks are represented in arrow form (the arrow connects the start and end point of each molecular trajectory, with the arrowhead indicating molecule direction). Molecular tracks are shown by coloured lines, with the colour indicating the time in the image sequence. Munc18-1 molecular accumulations in varicosities become disrupted in the presence of syntaxin1a Δ6, ^L165A, E166A^). Scale bar, 1 μm. Colour bar: start time, black; end time, white. The diffraction-limited image is shown in grey. (**b**) Density plot showing the number of molecular tracks crossing each pixel in the image, demonstrating munc18-1 accumulation in synapses decreased when interaction with syntaxin1a is disrupted and that the introduction of the mutant did not decrease munc18-1 molecular number. Colour coding shows the track number. Scale bar, 10 μm. (**c**) Left panel: diagrammatic representation of the Rose diagram calculations. Once a molecular direction is established, every angle between molecular trajectory steps (2–3′ or 2–3′′) is measured. Each munc18-1 angle (Ø′ or Ø′′) is then incorporated into circular histogram ‘Rose Diagrams’ (centre and right panels). In this analysis, each of the 36 segments represents a bin of 10° angle, segment magnitude represents the number of trajectories in each histogram bin and colours indicate the molecular track speed. Molecular reversals are thus shown as a leftward deflection, as molecules return from where they originated. These show an increase in reversing munc18-1 molecules and a specific decrease in the number of the slowest-moving molecules with a concomitant increase in the proportion of the fastest munc18-1 molecules when interaction with syntaxin1a is disrupted. (**d**) Reconstructing an image to illustrate where track reversals occur highlights a loss of munc18-1 directionality when interaction with syntaxin1a is disrupted. Green spots indicate complete track reversals; grey background represents the outline of the cells. Scale bar, 5 μm. (**e**) Quantifying molecular speeds shows that when binding with syntaxin1a is disrupted, there is a relative decrease in the proportion of slowest munc18-1 molecules and a concomitant increase in faster-moving munc18-1. Grey bars, munc18-1 molecule speeds in the presence of syntaxin1a Δ6, ^L165A, E166A^); black bars, control munc18-1 molecular speeds. Error bars=s.e.m.

To test this hypothesis directly, we quantified the mobility of individual munc18-1 molecules (as opposed to earlier bulk studies) in living neurons using state-of-the-art single-particle tracking approaches combined with PALM (sptPALM^12^,^13^,^24^). Large cohorts of single munc18-1 molecules (6,584 single molecules from *n*=3 independent experiments) were tracked, revealing kinetically and spatially distinct molecular populations (Fig. 2a–d). Consistent with the notion that the molecules are trafficked along processes before accumulation in synapses, munc18-1 molecules exhibited a restricted motion in puncta, whereas a separate population of munc18-1 molecules displayed directed movement, travelling with long displacements between synapses (Fig. 2a–d).

### Munc18-1 trafficking depends on binding to syntaxin1a

To determine whether the interaction with syntaxin1a affected these different behaviours, we introduced a dominant-negative mutant of the t-SNARE protein (Syx Δ6, ^L165A, E166A^-mCerulean^5^) that, in neuroendocrine cells, disrupts both munc18-1-membrane localization (as this is dependent on syntaxin1a interaction^25^,^26^) and exocytosis^10^,^14^. This mutant, in contrast to the syntaxin1a ‘open’ mutant (L165A, E166A (ref. 27) that has an unaltered affinity for munc18-1, has a nearly 10-fold reduced affinity *in vitro*^5^ as a result of a combined disruption of both ‘N-peptide’ and ‘closed-form’ interaction. It is important to note that this mutant still interacts with munc18-1 at least *in vitro*, and so any effects we observe likely point to a partial disruption of interaction as opposed to a complete abolition of binding. This approach allowed us to quantify munc18-1 molecular behaviours in detail in cells readily identifiable as containing mutant syntaxin1a by virtue of their cyan fluorescence. Comparing data from this system with that where we introduced wild-type (wt), full-length syntaxin1a as a control revealed that munc18-1 molecular trafficking was dependent on interaction with syntaxin1a; disruption of binding with syntaxin1a resulted, qualitatively, in a more uniform distribution of munc18-1 tracks (Fig. 2b). Quantifying munc18-1 track lengths revealed significantly longer movements when SNARE interactions were disrupted (~25% longer; 1.96±0.6 nm (mean±s.e.) when co-expressed with wt syntaxin1a, 2.45±0.5 nm in the presence of Syx Δ6, ^L165A, E166A^, *n*=1,613 and 3,605 tracks from three cells, respectively, *P*<0.05).

A major advantage of sptPALM, particularly if the most advanced tracking algorithms are used^12^,^13^,^28^, is the delivery of a vast amount of content describing the movements of large numbers of single molecules. The large samples and accurate data in turn offer statistical certainty and resolution much greater than can be achieved when using bulk studies. Taking advantage of this high-content data, and to further understand the effect of syntaxin1a interaction on munc18-1 molecular dynamics, we plotted every trajectory angle taken by every munc18-1 molecule, plotting in circular histograms, or ‘Rose Diagrams’^12^,^13^, to illustrate differences in trajectories and speeds (Fig. 2c). The Rose diagram is simply a circular histogram showing the direction taken between steps of a molecular track, with 36 bins of 10° angles adding to a circle of 360°. The size of each ‘pie piece’ reports the number of measurements in each histogram bin, with single-molecule speed shown as a coloured bar.

Using this approach, we wanted to determine whether munc18-1 molecule movements in living neurons were affected by the disruption of interaction with syntaxin1a. To attempt to measure this, we selected one parameter; the angle of every munc18-1 molecular movement relative to the direction of the preceding step in each track. This analysis reveals whether molecules diffuse freely, apparent as a completely round histogram (that is, with the same number of measurements in each 10° bin), or if they are they are somehow directed or constrained, evident as a skewing of the histogram in one preferred direction or another*—*so it becomes ovoid in shape. This detailed analysis found a decrease in the proportion of slowest-moving munc18-1 molecules and an increase in the fastest (Fig. 2c,e), when both interaction modes with syntaxin1a were disrupted. This speed change is indicative of a loss of interaction, as soluble munc18-1 molecules are predicted to move more quickly than those in a membrane-associated complex with syntaxin1a.

We also found a relative increase in munc18-1 trajectory reversals when syntaxin1a interaction was disrupted (5,602 molecular track reversals when interaction was disrupted, versus 2,416 when interaction was normal): this is apparent as a skew in the Rose diagram to the left, as the movement between trajectory steps is prone to be backwards.

This analysis alone does not allow a full interpretation: to understand these data, spatial information is required. Therefore, to interpret this complex behavior, we extracted the molecules that showed these predominant direction reversals and reconstructed these into an image representing their original position in the cell (Fig. 2d). This revealed that when both the available modes of interaction with syntaxin1a were disrupted by mutation to Syx Δ6, ^L165A, E166A^, munc18-1 molecules lost directionality throughout the entire cell, whereas in the presence of wt syntaxin1a, such molecular behaviours were less spatially homogeneous (Figs 1 and 2 and Supplementary Fig. 1). Finally, we plotted the same munc18-1 single-molecule velocities in a traditional histogram, reiterating that when syntaxin1a interactions were disrupted, molecular speeds increased (Fig. 2e). Together, these experiments show that munc18-1 molecular behavior and directionality, at least in part, depend on the interaction with syntaxin1a using one of the available binding modes.

The molecular speeds we measure are faster than those previously reported for these and other neuronal proteins using lower temporal resolution approaches^23^,^29^, possibly due to the under-sampling in earlier studies. To clarify this, we performed fluorescence recovery after photobleaching (FRAP) experiments at decreasing bleaching and sampling rates. Comparing this with molecular tracking at the same rates, and with fluorescence correlation spectroscopy (FCS) measurements (below) at microsecond rates, we found that apparent molecular speeds varied with acquisition rate down to ~3 Hz, at which point they plateaued, confirming that our sampling rates were sufficient to capture rapid molecular dynamics from synapses (Supplementary Fig. 2).

Together this combination of single-molecule resolution imaging, high-resolution tracking and new informatics approaches reveal that the behavior of munc18-1 in central neurons is controlled by an interaction with syntaxin1a. Which mode of interaction is utilized at the different neuronal sites identified using these approaches, and how differences in mode may be functionally important in neurons cannot be fully addressed using imaging alone; spectroscopy is required to begin to probe molecular interactions.

### Syntaxin1a–munc18-1 interactions in central neurons

Munc18-1 has been attributed a range of essential functions, as a chaperone^26^,^30^, as a ‘docking factor’^31^, acting with SNAP-25 and synaptotagmin^32^ and as an essential modulator of the very last stages of synaptic vesicle fusion^7^, even shaping fusion pore kinetics^8^. These distinct functions suggest a molecular interaction pathway between docking and fusion^3^, as it is accepted that if munc18-1 regulates the late stages of vesicle exocytosis, it must be via interaction with the N terminus of syntaxin1a (as opposed to ‘closed’ syntaxin interaction) in the ternary SNARE complex^6^. However, recent data using mutagenesis and electrophysiology showed that whereas munc18-1 has a role in the docking and priming stages of exocytosis, its continued association with the N-peptide of syntaxin1a appears dispensable for normal secretion^33^. What is still not known, however, is whether munc18-1 and syntaxin1a dissociate at all during synaptic exocytosis or whether they remain bound, and when if tested directly, the N-terminal mode interaction persists. Given that exocytosis is likely to be mediated by only a few SNARE/SM molecules^12^,^13^,^34^,^35^ that we have shown here that localize dynamically to synapses in an interaction-dependent way (Figs 1 and 2), single-molecule resolution approaches with very high temporal rates are required to dissect synaptic interactions.

**Figure 3:**
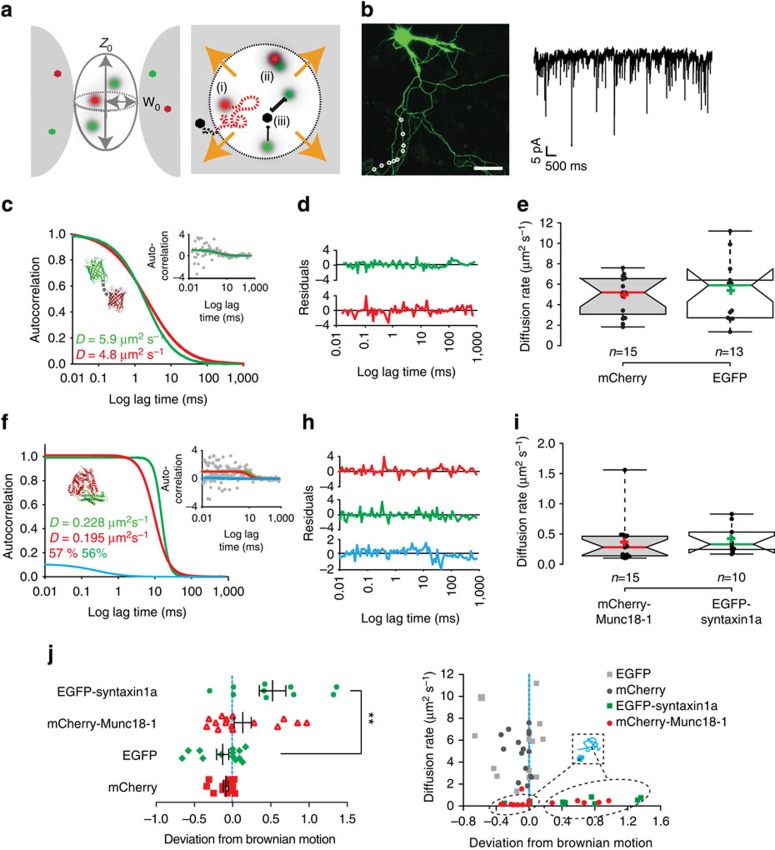
Syntaxin1a and Munc18-1 are in an immobile complex in resting synapses. (**a**) FCS and FCCS were used to probe syntaxin1a–munc18-1 interaction in synapses on a rapid timescale. The dimensions of the excitation spot (*W*_0_ and *Z*_0_) are determined experimentally using purified fluorescent proteins (Supplementary Fig. 3). FCS provides high spatiotemporal resolution of protein diffusion rate and mode (i) interaction (ii) and reaction kinetics (iii). (**b**) Representative cortical neuron transfected with munc18-1-EGFP (left panel) showing varicosities (white circles) where measurements were acquired. Scale bar, 5 μm. Example spontaneous mEPSC trace (right panel). (**c**) Representative autocorrelation fits of unfused EGFP and mCherry molecules in neuronal synapses, autocorrelation trace of the same data (insert). No cross-correlation could be detected. (**d**) Fit residuals of the data in **c**. (**e**) Box plot of EGFP and mCherry diffusion in resting synapses. (**f**) Representative autocorrelation and cross-correlation fit (blue) result of syntaxin1a (green) and munc18-1 (red), raw autocorrelation trace of the same data (insert). (**h**) Fit residuals of the data in **f**. (**i**) Box plot of syntaxin1a and munc18-1 diffusion data in resting synapses (simplified bar charts showing means and s.e.m. for each treatment are presented in Supplementary Fig. 3). The rates of diffusion were calculated from the derived autocorrelation curves; centre lines represent the median; cross indicates the mean; box limits indicate the 25th and 75th percentiles and whiskers extend to minimum and maximum points. Notches are 95% confidence intervals that two medians differ. There are no statistical differences between groups. (**j**) Graphical representation of the calculated deviation from Brownian motion of each sample diffusion data. EGFP and mCherry in synapses diffuse with a Brownian motion (indicated by the blue dashed line), indicative of free diffusion in the synaptic cytosol, whereas syntaxin1a and munc18-1 molecules deviate from this behavior, indicative of membrane anchoring and thus interaction (as monomeric munc18-1 is a soluble protein). In this plot (left), the central black bars indicate the median, with error bars indicating s.e.m. Right panel: plotting ‘Deviation from Brownian motion’ against diffusion rate reveals two populations of mCherry-munc18-1 behaviours in synapses; both populations have low diffusion rates but differ in diffusional behavior*—*one group of molecules diffuse in a directed manner and the other appears caged.

To examine dynamic molecular interactions, as opposed to co-localizations, in intact synapses, we used FCS that reports molecular concentrations, diffusion rates and interactions on the microsecond timescale. This has the advantage over more commonly used FRET assays in that it can report molecular dissociations as well as interactions, essential when analysing a dynamic molecular pathway (Fig. 3a). FCS was performed in electrically active cortical neuronal synapses in cells expressing fluorescent reporter molecules (Fig. 3b). To gain specificity and examine interactions at different points in the synaptic vesicle pathway, we employed botulinum neurotoxin (BoNT)-resistant molecular probes, expressed at very low levels (10–100s of molecules per measurement) as tracers, and specifically removed endogenous SNARE proteins as required to reveal interaction modes at defined points in the synaptic secretory pathway.

First, we tested whether we could quantify molecular diffusion rates and interactions (Fig. 3c–e) by expressing EGFP and mCherry fluorescent proteins, and performing FCS measurements. Protein expression was driven from a ‘crippled’ cytomegalovirus (CMV) promotor^36^ ensuring that tracer levels of heterologous proteins were present, with varicosities containing the lowest detectable fluorescence selected for analysis. Both the EGFP and mCherry photon fluctuations could be measured with a 2-μs acquisition rate allowing protein molecular concentrations to be measured *in situ*, ensuring similar expression levels in all experiments. FCS has the advantage of being applicable equally in cellular systems as well as *in vitro*, so we compared these data with those acquired from defined concentrations of highly purified fluorescent proteins *in vitro*; these calibrations confirmed that we could accurately report molecular behaviours, diffusion rates and concentrations in neurons (Supplementary Figs 3–5). Box plot data are also simplified and presented in Supplementary Fig. 4. Representative raw data and fitted autocorrelation functions are shown in Supplementary Fig. 5.

As our single-molecule imaging data suggested that munc18-1 behaviour in synapses was syntaxin1a dependent, we measured the rates of diffusion of mCherry-munc18-1 and EGFP-syntaxin1a in the varicosities identified by synapsin co-staining in earlier experiments, again acquiring data with an acquisition rate of 2 μs (Fig. 3b). Autocorrelation curves for these data accumulated over 5–10 s were generated, delivering similar diffusion rates for munc18-1 and syntaxin1a molecules (Fig. 3f–i; 0.35±0.09 μm^2^ s^−1^ and 0.41±0.07 μm^2^ s respectively; mean±s.e.m., *n*=at least 10 experiments). Importantly, munc18-1 diffused at a rate similar to that of syntaxin1a, suggestive of protein–protein interaction in the resting synapse (as munc18-1 is a soluble protein and syntaxin1a is an integral membrane protein). Closer inspection of the diffusional behavior of these proteins revealed that both diffused with directed motion, indicative of membrane association (Fig. 3j). To rule out the possibility that this was simply a result of the crowded synaptic microenvironment^37^, we performed similar FCS experiments using unfused (that is, soluble) EGFP in cortical varicosities, finding a significantly faster diffusion rate of, (5.32±0.83 μm^2^ s^−1^, mean±s.e.m., *n*=13, four cells, Mann–Whitney *U*-test, *P*<0.001) that followed a model of Brownian free diffusion (Fig. 3j).

Next to determine the rate of diffusion of monomeric munc18-1 molecules in the cellular environment, we first chose HEK293 cells, known not to express syntaxin1a or other munc18-1-binding proteins. Munc18-1 in this cellular expression system was cytosolic, and FCS autocorrelation analysis yielded a diffusion rate of 9.26±1.86 μm^2^ s^−1^ (mean±s.e.m., *n*=9 independent experiments; Supplementary Fig. 3). This molecular diffusion was slower than for cytosolic EGFP alone (*D*=19.66±1.06 μm^2^ s^−1^, mean±s.e.m., *n*=9 independent experiments), consistent with the molecular mass of munc18-1-EGFP being four times that of unfused EGFP, resulting in a slower diffusion rate. As synaptic munc18-1 had a significantly slower rate of diffusion than cytoplasmic munc18-1 in a HEK293, where no syntaxin1a is present, this provided further evidence of an interaction with syntaxin1a. Correlating diffusion rate with ‘Deviation from Brownian motion’*—*a parameter describing the molecular motions as they pass through the excitation volume^38^*—*revealed that two populations of munc18-1 molecules were detectable in synapses; both shared similar diffusion rates with syntaxin1a but differed in diffusional behaviour, suggesting that a small subset of munc18-1 molecules were associated with a complex in synapses distinct from a direct interaction with syntaxin1a. Notably here, no syntaxin1a was ever detected that did not have munc18-1 molecules sharing identical dynamics (Fig. 3j). As a final, additional test of protein–protein interaction, we cross-correlated the fluorescence fluctuation data (fluorescence cross-correlation spectroscopy; FCCS) acquired from mCherry-munc18-1 and EGFP-syntaxin1a molecular signals in synapses, finding that ~60% of each binding partner co-diffused in the synapse (Fig. 3f). Despite this result, this latter measure, FCCS, proved unreliable in our hands as it was technically difficult to cross-correlate data from the short recordings we required in small synapses. However, the multiple alternative parallel measures confirm that we could quantify, robustly, protein–protein interactions inside synapses with high temporal resolution and that the diffusion rates measured, combined with data describing molecular freedom, act as an accurate, rapid reporter for direct interactions.

A concern about these assays is that the background of endogenous syntaxin1a could interact with our mCherry-munc18-1 probe expressed at tracer levels, so skewing our data. To control for this and improve the specificity of these assays, we removed endogenous syntaxin1a from synapses by treatment with BoNT/C, which cleaves proteolytically syntaxin1a. As a test for endogenous syntaxin1a cleavage and to further test our hypothesis that munc18-1 interacts with syntaxin1a in the synapse, we determined the diffusion rate of mCherry-munc18-1 molecules in these cells. mCherry-munc18-1 in these samples had a significantly faster diffusion rate than in cells with intact syntaxin1a (diffusion rate in BoNT/C-treated cells=3.27±0.44 μm^2^ s^−1^, increasing from 0.35±0.09 μm^2^ s^−1^, mean±s.e.m., *n*=10, *P*<0.001). This rate was also slower than that we determined for cytosolic monomeric munc18-1 in HEK293 cells, indicative of the crowded synaptic environment^37^.

**Figure 4:**
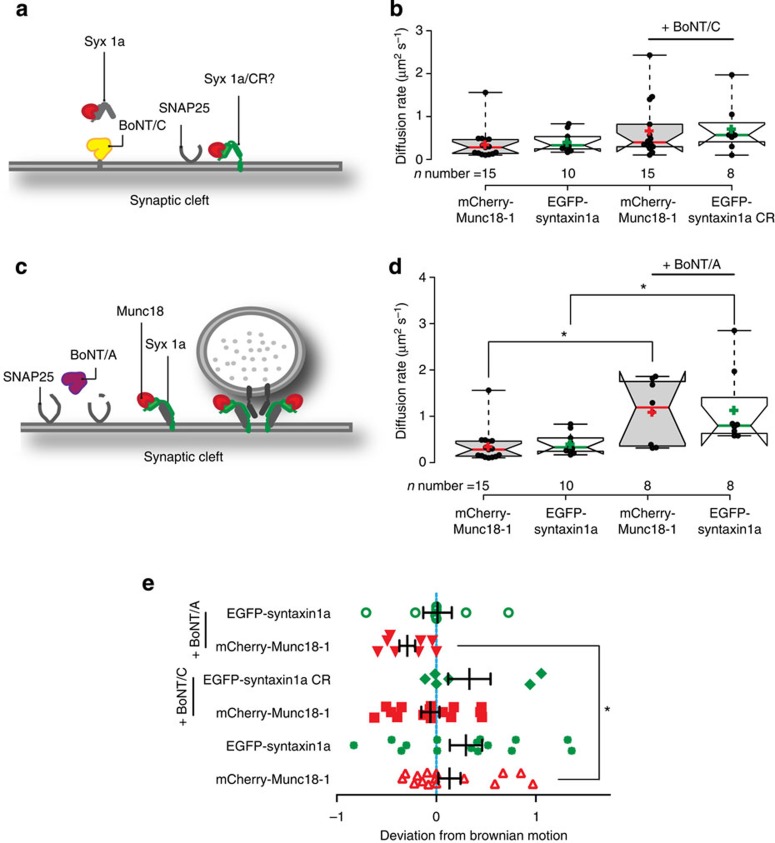
Munc18-1 interacts directly with syntaxin1a in the t-SNARE heterodimer. (**a**) Cartoon illustrating BoNT/C cleavage of endogenous syntaxin1a in a synapse. The question mark illustrates the experimental question: do munc18-1 and syntaxin1a interact in the presynaptic area. (**b**) Box plot comparing the diffusion rates of WT Munc18 paired with syntaxin1a or syntaxin1a/CR. Line indicates BoNT/C-treated neurons. Both protein pairs co-diffused with similar rates and toxin cleavage did not result in any significant changes. (**c**) Model illustrating BoNT/A cleavage of endogenous SNAP-25 in a synapse. (**d**) BoNT/A cleavage of SNAP-25 results in an increased diffusion rate of both munc18-1 and syntaxin1a compared with non-treated controls. Stars indicate statistical significance (Mann–Whitney *U*-test, *P*<0.05). (**e**) Syntaxin1a and munc18-1 in BoNT/A treated neurons co-diffuse at a similar rate and exhibit non-Brownian diffusion.

Next we introduced EGFP-syntaxin1a mutated to be BoNT C resistant^39^; (syntaxin1a-CR; Fig. 4a). Munc18-1 and syntaxin1a-CR colocalized with no gross spatial reorganization in these cells, imaged at diffraction-limited resolution. FCS studies as before confirmed that the two protein partners in this clean background co-diffused with statistically similar diffusion rates (Fig. 4b; 0.68±0.18 μm^2^ s^−1^ and 0.72±0.2 μm^2^ s^−1^, respectively)*—*furthermore, these behaviours were identical to those found in non-toxin-treated samples (see Fig. 3).

Thus, these combined experiments demonstrate that we can detect and quantify presynaptic protein interactions on the molecular level at the high speeds necessary to determine dynamics.

### Dissecting interaction modes during the vesicle fusion cycle

Having confirmed that we could detect the interaction in synapses, we next set out to determine whether the munc18-1 there interacted mainly with monomeric syntaxin1a, or with the t-SNARE heterodimer or the ternary SNARE complex. To address the first question, we treated samples with BoNT A (BoNT/A), which cleaves the carboxyl-terminal nine amino acids from SNAP-25, altering the t-SNARE heterodimer conformation^11^,^40^. Further, it is also known that specifically BoNT/A cleavage of SNAP-25 does not alter the affinity of the t-SNARE heterodimer partners^40^. Performing our FCS analyses as before, using tracer probes in synapses, revealed that both syntaxin1a and munc18-1 increased their diffusion rates by a similar extent compared with untreated synapses, consistent with being part of a complex with lower molecular mass (Fig. 4c,d). Furthermore, analysis of the mode of diffusion found that whilst syntaxin1a and munc18-1 remained in complex, they co-diffused differently after BoNT/A treatment, adopting a less restricted behaviour (Fig. 4e).

Together these data suggest that munc18-1 interacts with the t-SNARE heterodimer predominantly and not with monomeric syntaxin1a in resting synapses.

### Determining syntaxin1a-munc18-1 pre-fusion interaction mode

**Figure 5:**
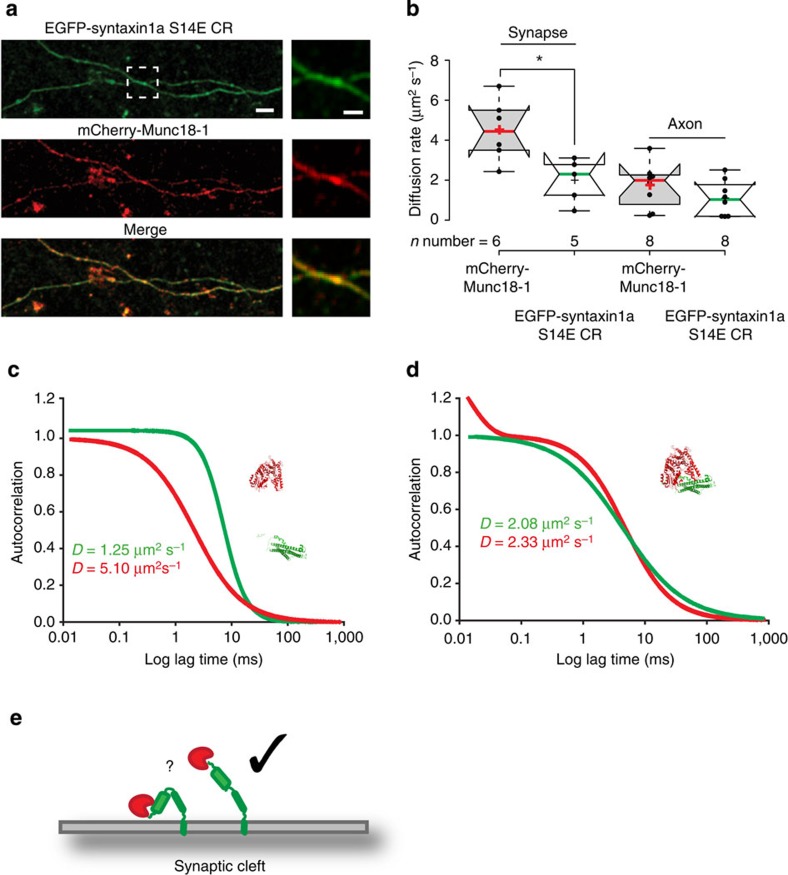
Munc18-1 interacts with syntaxin1a in varicosities via the syntaxin1a N-terminal peptide motif but can use alternative interaction modes in neuronal processes. (**a**) BoNT/C-treated neurons transfected with mCherry-munc18-1 and EGFP-Syntaxin1a^S14E^CR; both proteins have a localization not dissimilar from the non-treated mCherry-munc18-1-EGFP-syntaxin1a. Scale bar, 1 μm. The boxed area is zoomed in the right panels (scale bar, 1 μm). (**b**) Munc18-1 and syntaxin1a diffusion rates measured in synaptic regions. The diffusion rate of munc18-1 increased significantly compared with syntaxin1a^S14E^CR indicating that the two proteins no longer interact. In axons, the interaction between the two proteins is stabilized with both co-diffusing with an identical rate and mode. (**c**) Representative autocorrelation fits of mCherry-munc18-1 and EGFP-syntaxin1a^S14E^CR in synapses. The diffusion curve of munc18-1 no longer has a sharp decay indicating a switch in behaviour to free diffusion. (**d**) Representative autocorrelation fits of mCherry-munc18-1 and EGFP-syntaxin1a^S14E^CR in neuronal processes. Both proteins have similar rates and modes of diffusion. (**e**) Cartoon summarizing this finding: in the resting synapse, munc18-1 is predominantly associated with open syntaxin1a via the N-peptide interaction.

Syntaxin1a and munc18-1 can adopt functionally and spatially distinct modes of interaction^5^,^6^. As we found that munc18-1 interacts with the t-SNARE heterodimer, this suggests binding to the so-called N-peptide motif in syntaxin1a. To address this, we transfected cells with a phosphomimetic BoNT/C-resistant Syntaxin1a^S14E^CR. Syntaxin1a has a consensus phosphorylation site for Casein kinase II at Serine 14; 40% of total brain syntaxin1 was shown to be phosphorylated at this site in axons but not in active zones^23^; furthermore, we showed recently that charge alteration at this site destabilized specifically the interaction between the N-terminal peptide motif of syntaxin1a and munc18-1, whilst leaving the alternative modes of interaction between these partners intact^10^,^41^. As this mutant has a significantly lower affinity for munc18-1 than wt-syntaxin1a^10^, a (toxin resistant) construct was thus used in neuronal cells where endogenous syntaxin1a was removed using BoNT/C poisoning (see Fig. 4); EGFP-Syntaxin1a^S14E^CR and mCherry-munc18-1 co-localized with an arrangement not dissimilar from the endogenous protein patterns, with enrichment in synapses (Fig. 5a). FCS analysis was then employed to further understand the interaction between these proteins (as opposed to the co-localization, which is limited by the resolution of the imaging system (~250 nm in this case)). These experiments found that the rate of diffusion and restricted, membrane-associated behaviour of syntaxin1a^S14E^CR was indistinguishable from that of wt EGFP-syntaxin1a (Fig. 5b,d). Importantly, in these synapses, mCherry-munc18-1 followed a freely diffusing model with a rate of diffusion approximately four times faster than syntaxin1a^S14E^CR (Fig. 5b,c) but in neuronal processes, behaved in a manner indistinguishable from that when associated with wt syntaxin1a (Fig. 5b,d). Notably, when the diffusion rate was plotted against diffusional behaviour as before, complexes that behaved in a tightly caged manner, with a very restricted diffusion rate, were absent compared with when wt syntaxin1a was present (Fig. 5d).

Together these data indicate that S14 modification disrupts the interaction between syntaxin1a and munc18-1 and thus demonstrate that munc18-1 interacts predominantly with the N-terminal motif of syntaxin1a in resting synapses. Furthermore, a specific subpopulation of complexes in synapses, identified by virtue of simultaneously caged, non-Brownian and slow diffusion, was absent when syntaxin1a*–*munc18-1 N-terminal interaction was disrupted in synapses.

We hypothesized that this subpopulation of complexes, representing on an average ~10% of SNARE molecules, represented those ‘release-ready’ SNARE complexes with munc18-1 engaged that may go onto support synaptic vesicle fusion during an action potential. We tested this in two ways; first, as the association of the vesicular SNARE, synaptobrevin, is known to occur after t-SNARE heterodimer formation^42^, even in the presence of SNARE complex-associated munc18-1 (refs 5, 6, 43), we treated neuronal preparations with tetanus neurotoxin (TeNT) to cleave specifically synaptobrevin^44^. FCS after this treatment showed that in resting synapses, syntaxin1a and munc18-1 interacted as before but exhibited rates of diffusion in complex significantly faster than found in untreated synapses (Supplementary Fig. 6), suggesting that the munc18-1*–*syntaxin1a complexes we detected could be associated subsequently with synaptobrevin. The hypothesized ‘release-ready’ molecular complex diffusional behaviours we had previously observed, were absent after this synaptobrevin cleavage (Supplementary Fig. 7).

### Following synaptic protein interactions during exocytosis

We next wanted to determine the synaptic protein interaction pathway during depolarization and exocytosis. A recent study reported a large-scale, temporary dispersal of munc18-1 from synapses during electrical stimulation^23^, but it remains unclear whether all the munc18-1 redistributes from the synapse during stimulation or whether some remains associated with syntaxin1a and only soluble munc18-1 disperses. If the latter is true, then it is credible that the munc18-1 molecules that remain bound to syntaxin1a are contributing functionally to the synapse, whereas the soluble, mobile pool represents a reserve. Therefore, to determine whether neuronal activity resulted in a change in functional interaction (either dissociation or in mode of binding) between munc18-1 and syntaxin1a at the molecular level, we delivered trains of electrical depolarizations during FCS recording to determine on the molecular level whether interactions persisted. Parallel high-speed measurements (Supplementary Fig. 8a) of synaptic calcium levels and current-clamp electrophysiology found that repetitive neuronal action potentials were maintained for the time course of the stimulation with a mean time of ~5.4 s in these nerve terminals (Supplementary Fig. 8b). We established that these stimulation regimes induced depletion of synaptic vesicle pools using FM-dye assays, and that the presence of our probes at such low levels supported normal fusion kinetics (Supplementary Fig. 8c). Exocytosis continued for several seconds beyond voltage-gated Ca^2+^ channel inactivation, determined by using transfected pHluorin reporters in combination with bafilomycin blockade of vesicle reacidification (SypHy) assays (Supplementary Fig. 8d); these data indicated that Ca^2+^ concentration was sustained at levels sufficient to elicit vesicle fusion and that the expression of our fusion proteins had no effect on vesicular fusion kinetics, or the size of the RP or RRP, as compared with non-transfected controls. Maximal stimulation elicited a reported release of 50±10.2% of synaptic vesicles across all synapses*—*equating to ~100 synaptic vesicles per bouton. Thus, immediately on the initiation of depolarization for 30 s, we performed FCS measurements, to probe specifically the period within synapses when Ca^2+^ was maximal. No change was seen in the behaviours of unfused-mCherry or -EGFP that remained freely diffusing in synapses, confirming that the stimulation regime had no non-specific effect on the synaptic microenvironment or our assay (Supplementary Fig. 8e).

**Figure 6:**
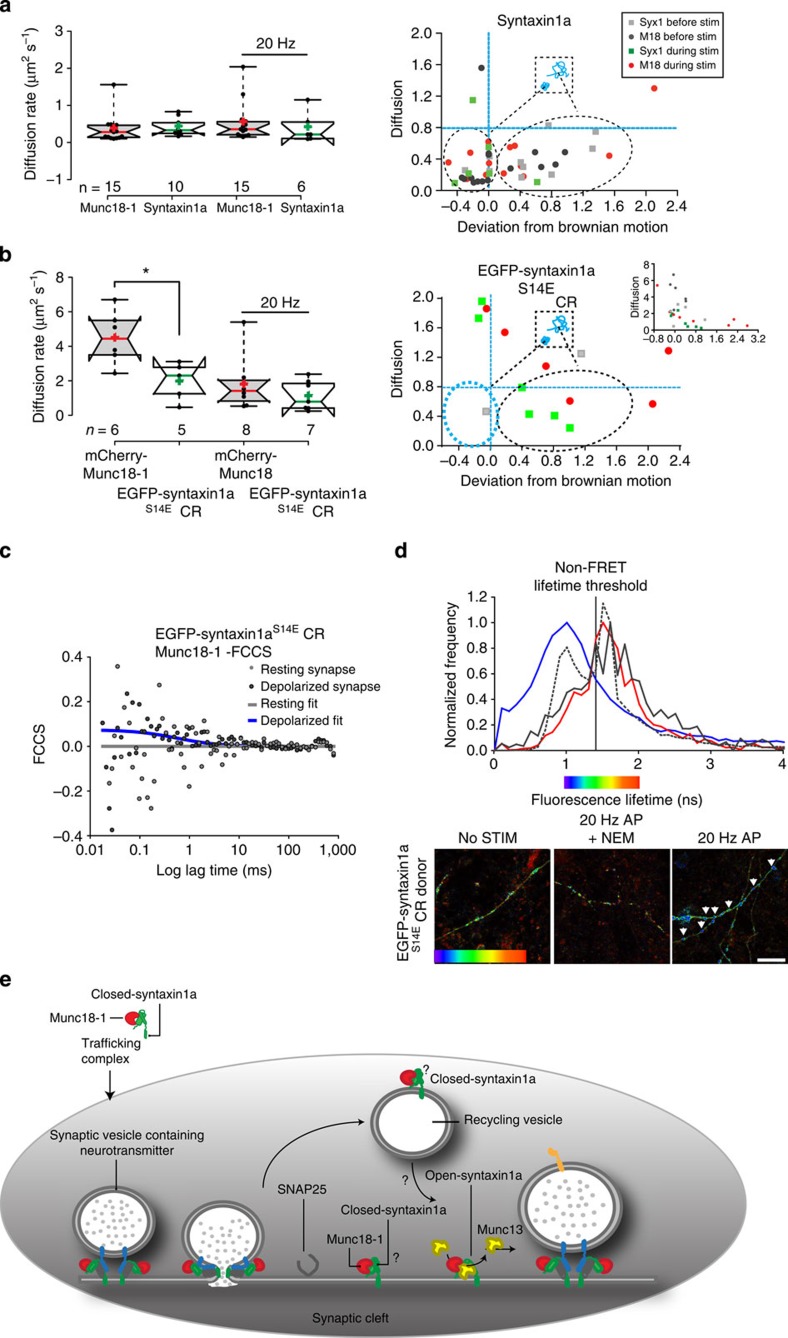
Syntaxin1a and munc18-1 interaction mode switches dynamically post exocytosis. (**a**) Left panel*—*FCS autocorrelation data show identical diffusion rates of syntaxin1a and munc18-1 in synapses before and during maximal stimulation. Right panel—Syntaxin1a and munc18-1 molecular diffusion models differ before and during maximal stimulation, with a 100% enrichment of molecular complexes exhibiting caged motion and low diffusion rates during stimulation (lower left quandrant of graph). (**b**) Destabilizing the N-peptide interaction results in a dissociation of syntaxin1a and munc18-1 specifically in synapses but a rapid interaction mode switch occurs during maximal stimulation. Left panel—FCS autocorrelation data deliver significantly different diffusion rates for munc18-1 and syntaxin1a in resting synapse, that converge during depolarizations. Right panel*—*EGFP-syntaxin1a^S14E^CR and munc18-1 molecular diffusion during maximal stimulation: munc18-1 molecules significantly slow during stimulation. Inset: expanded axes scales showing munc18-1 and EGFP-syntaxin1a^S14E^CR diffusion rates and modes in resting synapses. Symbols are as for **a**. Notably, the slow, caged population of molecules is absent during stimulations, in contrast to the wild-type syntaxin1a system. (**c**) Representative FLIM analyses of FRET between wt EGFP-syntaxin1a (donor) and mCherry-munc18-1 (acceptor); molecular interaction was reported as a mean donor fluorescence lifetime shorter than the mean non-FRET control across neurons (blue line); this threshold (solid vertical line) did not alter during maximal stimulation. Reduced FRET was detected between EGFP-syntaxin1a^S14E^CR and mCherry-munc18-1 before stimulation (red line) but this increased significantly after maximal stimulation (dashed grey line). This induction of interaction was abolished in the presence of NEM to inhibit SNARE disassembly post exocytosis (grey line). (**d**) FLIM maps illustrating spatial restriction of syntaxin1a^S14E^CR*–*munc18-1 interactions to varicosities after maximal stimulation are dependent on SNARE disassembly. Colour bar indicates donor fluorescence lifetime*—*shorter (blue) values indicate FRET and direct protein–protein interaction. Scale 1 ns (blue)*—*2 ns (red). Scale bar, 5 μm. (**e**) Cartoon incorporating our data into a refined model of the syntaxin1a*–*munc18-1 interaction pathway.

FCS experiments during depolarization showed identical diffusion rates and behaviours for both syntaxin1a (1.18±0.37 μm^2^ s^−1^) and munc18-1 (0.78±0.2 μm^2^ s^−1^, *n*=at least 10 independent experiments, mean±s.e.m.), which did not alter from the resting state, with both showing a restricted diffusion pattern indicative of a persistent interaction during depolarization (Fig. 6a). Importantly, there was an enrichment of ‘release-ready’ complexes during depolarization (to ~20% of detected molecules, compared with ~10% in resting synapses), lending weight to our conclusion that we could identify the subset of complexes active in synaptic vesicle exocytosis in these boutons (Fig. 6a).

### Syntaxin1a interaction switch occurs after SNARE disassembly

Next we performed experiments using the syntaxin1a^S14E^CR mutant, which we found previously not to interact with munc18-1 in resting neurons. Surprisingly, during depolarization, an induced interaction could be detected, with syntaxin1a remaining membrane associated and the previously fast, Brownian diffusion of munc18-1 in syntaxin1a^S14E^CR synapses slowing to rates identical to syntaxin1a^S14E^CR with a restricted motion (Fig. 6b). This effect was confined to synapses; measurements from axonal or dendritic regions of the cells showed no interaction between syntaxin1a^S14E^CR and munc18-1 under any circumstance. These experiments indicate that whereas in resting synapses the N-peptide interaction predominates, during depolarization and exocytosis, a molecular switch to munc18-1 interacting with closed-form syntaxin1a occurs. However, when we plotted diffusion rate against ‘deviation from Brownian motion’ as before (Fig. 6b), our measure that we postulated could identify ‘release-ready’ syntaxin1a*–*munc18-1 complexes, we did not find the slow-moving, caged complexes apparent previously (see Fig. 6a), presenting a conundrum.

Our maximal stimulation regime (20 Hz, 30 s) contains vesicle recycling and SNARE complex disassembly within this time; earlier work using identical regimes characterized the onset of endocytosis that increased to maximal rates within this 30-s period^45^. We hypothesized that the switch from fusion-permissive munc18-1 N-peptide interaction to inhibitory closed-syntaxin1a interaction occurs on the generation of monomeric syntaxin1a after exocytosis and SNARE complex disassembly. This hypothesis is further strengthened by our observation that this toggle occurs only where vesicle exocytosis is situated*—*that is, in synapses but not in neuronal processes (Fig. 6b–d). To support this, we separated our FCS data into two segmented temporal blocks; the first 10 s of stimulation (where we already determined that the voltage-gated Ca^2+^ channels are open and Ca^2+^ concentrations in the synapses are rising rapidly to maximal levels; Fig. 6b,c), and the final 10 s, after the majority of synaptic VGCCs have inactivated and recycling has commenced. Examining these data in this manner confirmed that indeed munc18-1 and syntaxin1a^S14E^CR do not interact at the initiation of depolarization but that a rapid induction of interaction, reported as a simultaneous decrease in the rate of diffusion of both munc18-1 and syntaxin1a^S14E^CR as well as FCCS, occurs after peak exocytosis (Supplementary Fig. 9).

To further test this over fields of synapses, we next performed FLIM^46^,^47^, to quantify FRET between EGFP-syntaxin1a (or EGFP-syntaxin1a^S14E^CR) and mCherry-munc18-1 (Fig. 6c,d), finding that as expected, syntaxin1a^S14E^CR and munc18-1 did not interact in synapses before depolarization, but that an induced interaction was strongly evident in varicosities after maximal stimulation. Finally, we conducted similar experiments in the presence of NEM to inhibit NSF and so SNARE complex disassembly post exocytosis^48^,^49^; in this condition, no induced interaction between munc18-1 and syntaxin1a^S14E^CR could be detected (Fig. 6c,d). Notably, the FLIM data describing syntaxin1a^S14E^CR*–*munc18-1 interaction are bimodal, indicating interaction dichotomy. Generating FLIM maps reconstructing these FRET data into an image revealed that syntaxin1a^S14E^CR*–*munc18-1 interactions post exocytosis are spatially restricted to varicosities, with no FRET detected in axons (Fig. 6d). These findings are summarized in a cartoon (Fig. 6e).

## Discussion

Visualizing and quantifying synaptic biology on the single-molecule level is required if we are to better understand how central synapses, and membrane trafficking processes in general, function. Here we present quantitative data describing the numbers, movements, localizations and interactions of presynaptic SNAREs and an important accessory protein, munc18-1, in active cortical neurons. SM protein biology has long been controversial; munc18-1 was originally thought to be an inhibitory factor, as it was isolated by virtue of its high-affinity interaction with monomeric syntaxin1a, sequestering it in an inactive form^50^. Contemporaneous findings that munc18-1 acted at the latest stages of vesicle exocytosis proved controversial^8^, but later it emerged that munc18-1 could indeed interact, via a different binding site in syntaxin1a, with the ternary SNARE complex^6^,^47^. Importantly, however, the precise temporal sequence of interactions between the SNAREs and regulators in the run up to synaptic vesicle fusion remains speculative, not least because of a lack of suitable approaches to probe directly molecular localizations, movements and interactions *in situ*. We present several independent lines of evidence to support the hypothesis that munc18-1 molecules are localized to synaptic terminals, in a syntaxin1a-dependent manner, where munc18-1 diffuses in a restricted fashion as compared to non-synaptic compartments. Trafficking along axons to the synaptic regions involved munc18-1 bound to ‘closed’ syntaxin1a, whereas on arrival at the synapse, syntaxin1a adopts an ‘open’ conformation and munc18-1 remains associated with the N-peptide motif. Our experiments using BoNT/A and TeNT provide data to suggest that at this stage, the majority of munc18-1 in the resting synapse is associated with syntaxin1a that is engaged in the ternary complex^7^. During depolarization and synaptic vesicle exocytosis, no large-scale dissociation of munc18-1 and syntaxin1a was detected. However, further dissecting this interaction in a clean background, using syntaxin1a^S14E^CR, that does not support N-peptide interaction with munc18-1 (ref. 10) revealed that a rapid switch occurs in binding mode after exocytosis. We conclude that a likely hypothesis is that the binding of munc18-1 to monomeric syntaxin1a in a closed form is induced post fusion after the ternary complex disassembly by alpha-SNAP and NSF and indeed, inhibiting disassembly with NEM also abolished the molecular toggle.

The data we present here provide direct and detailed information about the syntaxin1a*–*munc18-1 interaction pathway from trafficking, synaptic localization, interactions leading up to, during and after exocytosis. We hypothesize that a key role for munc18-1 is to prevent highly reactive, monomeric neuronal syntaxin1a from entering into SNARE complexes at the wrong point in the synaptic vesicle cycle. This hypothesis is attractive for several reasons; first, our earlier work in neuroendocrine cells showed directly that a principal role of munc18-1 is to prevent ectopic interaction between syntaxin1a and SNAP-25 in intracellular locations^26^. Second, to our knowledge, no data have ever been reported that show monomeric syntaxin1a in a cellular context. Our model here suggests that the syntaxin1a*–*munc18-1 equilibrium in the pre-synapse is normally shifted towards interaction, either in a complex that would permit SNARE complex formation, or in an alternative-binding mode known not to permit reactivity with SNAP-25 (refs 3, 6, 47). Earlier studies could not elucidate at which point in the exocytotic pathway the necessary switch in interaction mode occurred; our findings here help unify a number of apparently conflicting studies that variously found munc18-1 N-peptide interaction essential, or not required for normal synaptic function^3^,^4^,^7^; in any case, the role of the N-peptide interaction remains unclear^51^. We hypothesize that the N-peptide interaction in part is to maintain munc18-1 at a proximal location to the SNARE complex, ready to adopt the ‘closed-conformation’ interaction as rapidly as possible on the generation of monomeric syntaxin1a after vesicle fusion.

This is supported by our data here, which show that munc18-1 binds to closed (that is, non-reactive) syntaxin1a whilst trafficking along neuronal processes to the synapse, at which point the interaction mode alters, allowing munc18-1 to remain associated with the binary and ternary SNARE complex, pre-fusion. Our experiments showing a spatial and functional segregation of interaction mode, dissected using a phosphomimetic mutant of syntaxin1a, suggest, but do not show definitively, that phosphorylation of syntaxin1a near the N-peptide motif alters the preferred binding mode with munc18-1. If, as has been advocated recently, the ‘default’, lowest-energy interaction mode is for ‘closed-form’ binding with munc18-1 (ref. 51), then disruption of N-peptide interaction, as we previously showed^10^, via charge alteration on syntaxin1a Serine 14, long recognized as a site of syntaxin1a phosphorylation specifically in synapses (with no function identified)^22^, presents a simple mechanism for switching interaction mode.

How could the closed-form interaction be ‘opened’ in the pre-synapse, readying syntaxin1a–munc18-1 for subsequent rounds of fusion? Munc13-1 is localized to the presynaptic active zone, and its action there would increase either the availability of, or the affinity of syntaxin1a for SNAP-25 to induce this interaction switch with munc18-1 (ref. 52). During exocytosis, munc18-1 remains associated with syntaxin1a via the N-peptide motif throughout synaptic depolarization, perhaps with some functional input to shape the final fusion event^8^; in any case, this interaction ensures the close co-localization and preferential stoichiometry of munc18-1 with syntaxin1a. Immediately post fusion, the ternary SNARE complex is disassembled by the concerted actions of NSF and alpha-SNAP^53^, releasing reactive, monomeric syntaxin1a that can be immediately clamped by closely-associated munc18-1, remaining in the closed-conformation until the action of munc13-1 and SNAP-25 at the appropriate spatiotemporal location in the cycle (Fig. 6e).

Super-resolution microscopy has the potential to revolutionize our understanding of cell biology, by allowing the localization of single molecules with huge certainty in the environments in which they normally reside*—*inside cells. Molecular maps alone, however, could only rarely provide the information required to provide new models and understanding; tracking single-molecular behaviours, particularly powerful when large numbers of molecules can be sampled, provides a huge content not previously accessible. No super-resolution microscopy can detect protein–protein interactions *in situ*; the only way to quantify dynamic interactions at the molecular level required is to use spectroscopic approaches such as FLIM and FCS, presented here. The combination of these approaches is particularly powerful, serving to highlight not only the synergy between them, but also the limitations of each in isolation. Together these quantitative, single-molecule resolution approaches provide new data help define the entire lifecycle of the neuronal syntaxin-SM protein interaction and provide new insights into the synaptic vesicle cycle and the phenomenon of membrane fusion in general.

## Methods

### Primary cell culture, plasmids and transfections

Primary embryonic cortical neurons were prepared from the cortices of E18.5 embryos of Sprague–Dawley rats, which were killed according to Home Office Schedule 1 regulations. Cortical tissue was dissected and digested in papain (10 U ml^−1^) at 37 °C for 20 min. The tissue was then disaggregated and plated onto poly-D-lysine (50 μg ml^−1^)/laminin (10 μg ml^−1^)-coated cover slips at a density of 10^7^ cells ml^−1^. Cells were maintained in clear neurobasal medium (Invitrogen) supplemented with B27, glutamine and 1% pen-strep. Plasmids expressing munc18-1, syntaxin1a, syntaxin1a (openΔ6), syntaxin1a-CR, syntaxin1a^S14E^CR, synaptophysin-pHluorin and synapsin were transfected on DIV 12 with Lipofectamine 2000 (Invitrogen). Crippled mammalian expression plasmids were generated for munc18-1, syntaxin1a, syntaxin1a-CR, syntaxin1a^S14E^CR by replacing the CMV promoter with a truncated CMV.

### Botulinum neurotoxins

BoNT/A and BoNT/C were purchased from Miprolab, Germany. TeNT was purchased from Sigma-Aldrich. Neuronal cultures were exposed to 10 nM of toxin for 5 min at 37 °C in the presence of 55 mM KCL stimulation buffer, following this the cells were thoroughly washed and maintained in preconditioned neurobasal medium at 37 °C for a minimum of 2 h before commencing FCS experiments.

### Protein purification (EGFP, mCherry)

Coding sequences for EGFP (Clontech pEGFP-N1) and mCherry (Clontech pmCherry-N1) were amplified by PCR with added poly-histidine tag and restriction sites. PCR fragments were then inserted into the bacterial expression plasmid pGEX-KG for Glutathione-*S*-transferase (GST) fusion. EGFP fused to mCherry was created by ligation of EGFP into EcoRI and SalI sites of the newly constructed pGEX-KG_mCherry plasmid. Recombinant fluorescent proteins were expressed in *E.coli* and purified using affinity, ion exchange and size exclusion chromatography. Affinity chromatography was carried out using GST and His-tag purification. GST purification was performed first by incubation of clarified bacterial lysate with Glutathione-Sepharose beads (GE Healthcare). Beads were washed twice with 500 mM NaCl in buffer A (20 mM Tris pH 7.4, 1 mM EDTA and 0.1% TX-100) and twice with 150 mM NaCl in buffer A. Final washing step was done in low salt buffer A excluding EDTA followed by thrombin (Sigma) cleavage to elute the bound protein. His-Tag purification was performed on a HiTrap FF nickel chelating column (GE Healthcare) equilibrated with 20 mM imidazole in buffer B (20 mM Tris pH 7.4 and 150 mM NaCl) followed by a gradient elution with 500 mM imidazole in buffer B. The His-tag-eluted proteins were further purified by ion exchange using a Mono Q 5/50 GL column equilibrated with 25 mM NaCl in 20 mM Tris pH 8.5 and eluted with 1 M NaCL in 20 mM Tris pH 8.5. Gel filtration was carried out on a HiLoad 16/60 Superdex 200 column equilibrated with buffer A. All fractions were analysed by SDS–polyacrylamide gel electrophoresis and Coomassie staining, fractions containing fluorescent proteins were pooled and concentrated with a Vivispin 15R (Sartorius Stedim) before proceeding to subsequent purification steps. Protein concentration was measured using a Nanodrop A 280 module with entered values for protein molecular weight and molar extinction coefficient.

### Single-molecule imaging

All cortical neurons were transfected with either munc18 or syntaxin1a fused to PAmCherry. All PALM and dSTORM experiments were performed using an Olympus IX-81 microscope equipped with Olympus Cell^R acquisition software, an ImageEM EM-CCD 512 × 512 camera (Hamamatsu UK) and an Olympus × 150 UAPO 1.45NA oil lens with a resulting pixel size of 106 nm. For dSTORM, endogenous proteins were immunolabelled, after 90 min fixation in 4% (w/v) buffered paraformaldehyde with primary antibodies (munc18-1 (BD), syntaxin1a (HPC-1)). Both immunodetected munc18-1 and syntaxin1a were subsequently labelled with Alexa-647-conjugated anti-IgG (Invitrogen).

TCSPC measurements were made under 800–820 nm two-photon excitation, which efficiently excited cerulean without any measurable excitation or emission from EYFP, using a non-descanned detector (R3809U-50) multichannel plate-photomultiplier tube or a fast photomultiplier tube (H7422; both Hamamatsu Photonics UK) coupled directly to the rear port of a Zeiss LSM-510 Axiovert microscope. Images were recorded at 256 × 256 pixels from a 1,024 × 1,024 image scan with 256 time bins over a 12-ns period^54^. Off-line FLIM data analysis used pixel-based fitting software (SPCImage, Becker & Hickl). During all FRET experiments, neurons were bathed in phenol-free supplemented NBA (as described above) and maintained at 37 °C in 5% (v/v) CO_2_, 95% (v/v) air in a POC chamber (LaCon).

### mEPSC recordings

Cortical neuron cultures were washed and maintained in ‘stimulation’ buffer (136 mM NaCl, 2.5 mM KCl, 10 mM glucose, 10 mM HEPES (pH 7.4), 2 mM CaCl_2_, 1.3 mM MgCl_2_). Individual neurones were identified by light microscopy and the cell bodies subjected to whole-cell voltage clamp or current clamp using a HEKA EPC7 amplifier (HEKA Elektronik Lambrecht/Pfalz Germany). Data were acquired using ‘Clampex’ and analysed using Clampfit (Molecular Devices, LLC, CA, United States, version 10.3). Recordings were made at 18–20 °C using AgCl_2_ electrodes and filamented borosilicate glass patch pipettes with resistances of 3–7 MΩ. The bath solution contained stimulation buffer and the pipette solution contained (mM); 100 K-Gluconate, 20 KCl, 1 CaCl2, 1 MgCl_2_, 10 HEPES, 3 Phosphocreatine Na, 11 EGTA-KOH, 9 D (+)-Sucrose, 4 ATP-Mg_2_; pH 7.2; 295 mOsm kg^−1^. Cells were judged suitable for recording if the holding current at −60 mV holding potential was less than −100 pA. Voltage clamp was recorded in 10 mV steps from −70 to +80 mV every 2 s. To mimic bath stimulation, cells were current clamped to −60 mV and stimulated at 20 Hz for 60 s.

### FCS and neuronal stimulation

All FCS recordings were acquired using a Leica SP5 SMD confocal microscope using a × 63 1.2NA HCX PL Apo water lens and 488 or 561 nm CW lasers. Photon fluctuation data routed through a Picoquant PRT 400 router were acquired at microsecond rates using external Single Photon Avalanche Photodiodes (MicroPhoton Devices, Italy). Neurons were placed in a custom-made chamber with embedded platinum wires to deliver a stimulus. Cells undergoing electrical stimulation were immersed in stimulation buffer (136 mM NaCl, 2.5 mM KCl, 10 mM glucose, 10 mM HEPES (pH 7.4), 2 mM CaCl_2_, 1.3 mM MgCl_2_) and stimulated at 20 Hz for 30 s allowing all FCS data to be acquired during the stimulation train.

### FCS calibration

Before each FCS experiment, the values for *κ* (the ratio of the axial and waist excitation spot dimensions) and *V*_eff_ were determined using 10 nM Atto488 and Atto561 (Atto-Tec, Germany) standards in water at either 25 or 37 °C (Supplementary Fig. 3). Atto488 has a well-established diffusion rate of 400 μm^2^ s^−1^ (Picoquant). The resulting *V*_eff_ and *K* were verified using 10 nM purified EGFP and mCherry proteins (25 kDa) and 10 nM fused EGFP-mCherry protein (50 kDa) in 150 mM NaCl, 20 mM Tris pH 7.4, 1 mM DTT and 0.1% (v/v) Tween 20. The resulting diffusion coefficients of the fluorescent proteins (EGFP and mCherry of 124±0.17 and 106±0.12 μm^2^ s^−1^ at 37 °C, respectively; *n*=10) are consistent with Stokes–Einstein-estimated diffusion coefficients of 25 kDa proteins under these conditions. Calibration recordings of 30 s were made for each standard. These calibrations determined that the effective volume of the FCS spot was 0.29±0.04 μm^3^ at 37 °C; all *in cellulo* FCS measurements were made at this temperature. This volume is significantly smaller than the volume estimated recently for central neuronal synaptosomes using electron microscopy (0.37 μm^3^; ref. 37).

### FCS analyses

Autocorrelation traces were generated from the photon-counting histograms for each 5 to 30 s measurement using SymPhoTime v5.4.4 software (Picoquant, Germany). *In vitro* calibration traces were fitted using the Triplet model (three-dimensional free diffusion model with triplet state) with informed diffusion values to yield *V*_eff_ and *κ* values. Neuronal autocorrelation traces were fitted using a Triplet Extended model (two-dimensional anomalous diffusion model with triplet state), this model is designed for fluorescent molecules moving within a plane, for example, proteins in a membrane. Diffusion within the cells is expected to be anomalous; therefore, the anomaly parameter was not fixed to one. The anomaly parameter (*α*) measures the departure from free Brownian diffusion (*α*=1) to either superdiffusion (*α*>1) or subdiffusion (α<1) for a diffusing species. Autocorrelation curves with (*α*>1) display the sharpest decay, whereas the those with *α*<1 decrease quite slowly^55^.

### FRAP measurements and analysis

Photobleaching was carried out in total internal reflection fluorescence mode at 37 °C using the Olympus Cell^FRAP hardware attachment in conjunction with the Olympus Cell Excellence total internal reflection fluorescence system. A circular bleach area of radius 0.742 μm was selected and bleached between frames 5 and 6 of 50, taken every 31 ms, giving an acquisition rate of 32.3 Hz. Image J was used to extract intensity data from the resulting image files. These data consisted of the mean intensity with three circular regions of the image (*r*=0.742 μm)*—*one (*I*_frap_) centred on the bleached region, one outwith the cell or sheet (*I*_back_) and one (*I*_ref_) in a region of the membrane remote from the bleach point. *I*_norm_ for each frame was calculated by correcting the *I*_frap_ value using the *I*_ref_ data, to account for the general photobleaching that occurs during acquisition, as well as normalizing to the pre-bleach values, according to equation 1 below:


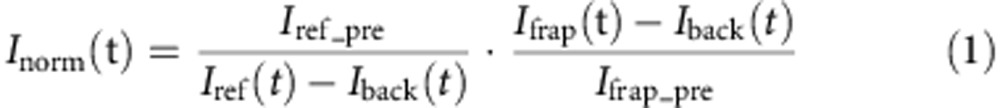


where *I*_norm_(*t*) is the normalized intensity; *I*_frap_(*t*) the measured average intensity inside the bleached spot; *I*_ref_(*t*) the measured average reference intensity; and *I*_back_(*t*) the measured average background intensity outside the cell. Subscript_pre means the averaging of intensity in the corresponding region of interest (ROI) before bleach moment after subtraction of background intensity.

Following the normalization of the data, the diffusion rate associated with the half-time of recovery for each curve was determined by fitting either a single-mode hyperbolic model (see equation (2), in which *y*=normalized intensity at time *t*, *y*_0_=normalized intensity at point of bleaching, *a*=maximal recovered intensity and *b*=*t*_1/2_, the half-time of recovery of the curve) or a single-mode exponential model (see equation (3) in which *y*=normalized intensity at time *t*, *y*_0_=normalized intensity at the point of bleaching, *a*=maximal recovered intensity and *b*=ln(0.5)/*t*_1/2_, the half-time of recovery of the curve).


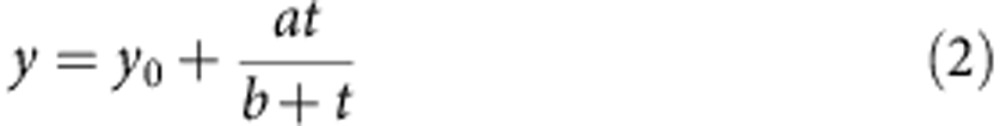



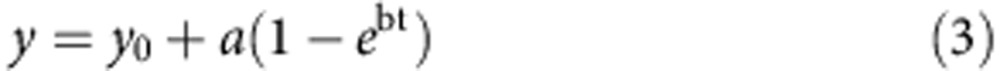


The half-time of recovery of these FRAP recovery curves is dependent on the diffusion rate, as is described in the standard relationship shown in equation (4).


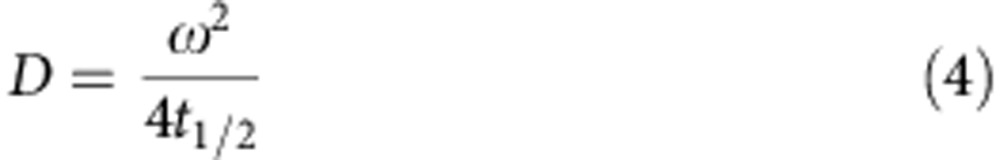


Fits were performed using the software package Sigma-Plot 12.5, and further calculation carried out using standard spreadsheet software (Microsoft Excel).

Different rates of acquisition were modelled by taking every second frame (modelling 16.1 Hz), every fifth frame (for 6.5 Hz), every tenth frame (3.2 Hz) or the frames acquired closest to 0.5 and 1 s intervals from the start of the time lapse for 2 and 1 Hz sampling respectively, and repeating the fitting on these data sets.

### Fluorescent monitoring of synaptic vesicle fusion

For experiments using FM1-43, cortical neurones that were transfected with both munc18 and syntaxin1a were loaded with 10 μM FM1-43 using a maximal stimulus of 50 mM KCl in stimulation buffer. After dye washout, cultures were mounted in a Warner imaging chamber with embedded parallel platinum wires (RC-21BRFS) and placed on the stage of Zeiss Axio Observer D1 epifluorescence microscope. Cultures were then subjected to a train of 60 action potentials (30 Hz) to unload the readily releasable pool and then two trains of 400 action potentials (40 Hz) to unloading the remaining vesicles in the reserve pool. Images were acquired using a Hammamatsu Orca-ER CCD camera at 480 nm excitation and >515 emission.

For sypHy experiments, transfected neurons were visualized using the same optical conditions as for the FM1-43 experiments. Cultures were stimulated with a train of 600 action potentials delivered at 20 Hz during continuous perfusion with stimulation buffer containing bafilomycin A1 (1 μM) to inhibit vesicle reacidification.

### Statistical methods

Statistical analyses were performed using Sigma-Plot v12.0. Data sets were first tested for normality using the Shapiro–Wilks test. Data that fitted a normal distribution were tested for statistical significance by two-tailed unpaired Student’s *t*-test. Failing normality, the data were analysed using the Mann–Whitney Rank-Sum test. All data presented as mean±s.e. Boxplots were created using the open source BoxPlotR tool as described in ref. 56.

## Author contributions

D.M.K., A.M.S., K.J.M., A.D., E.R.B., S.G., K.J.S., C.R. and R.R.D. acquired and analysed the data. R.S. and W.L. analysed the data. L.H.C., M.A.C., C.R. and R.R.D. designed the experiments. R.R.D. wrote the manuscript.

## Additional information

**How to cite this article:** Kavanagh, D. M. *et al*. A molecular toggle after exocytosis sequesters the presynaptic syntaxin1a molecules involved in prior vesicle fusion. *Nat. Commun.* 5:5774 doi: 10.1038/ncomms6774 (2014).

## Supplementary Material

Supplementary Information Supplementary Figures 1-9.
