# Supplementary material for: A molecular toggle after exocytosis sequesters the presynaptic syntaxin1a molecules involved in prior vesicle fusion
Source: Nat Commun. 2014 Dec 17;5:5774. doi: 10.1038/ncomms6774 (PMC4284649; doi:10.1038/ncomms6774)
Supplement: Supplementary Information — Supplementary Figures 1-9. [file ncomms6774-s1.pdf]

Supplementary Figure 1

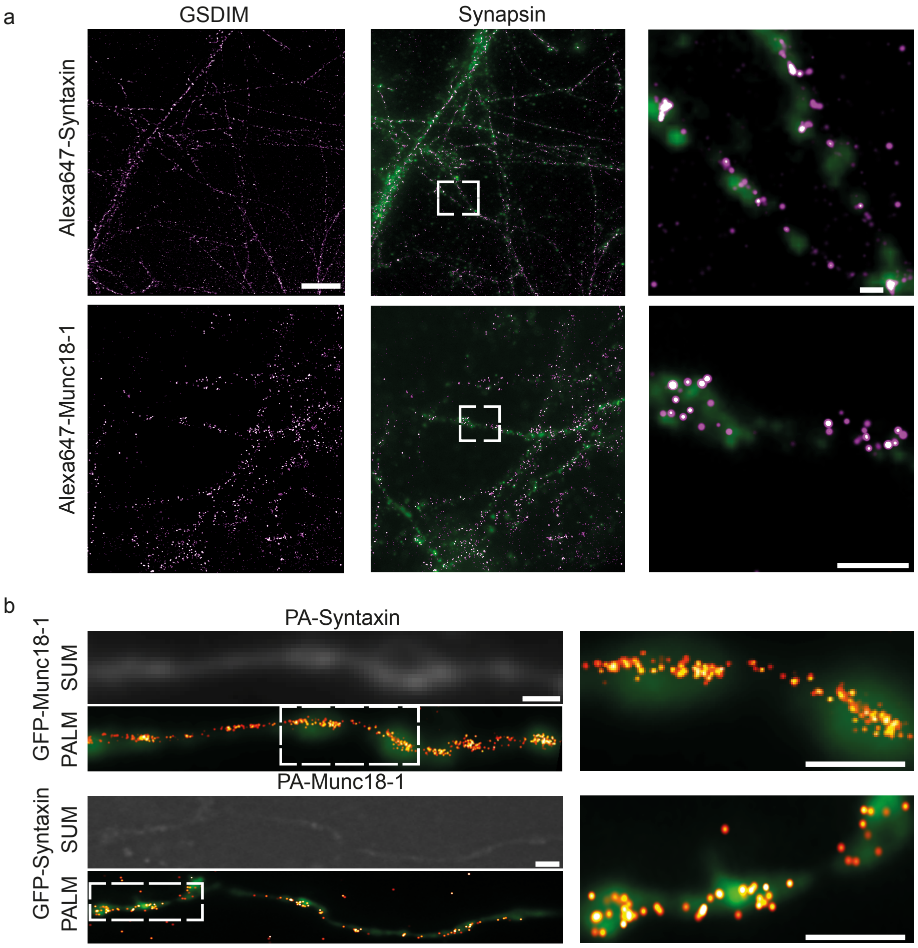

Munc18-1 and syntaxin1a molecules co-accumulate in synapsin-positive synapses
a GSDIM map of immunolabelled munc18-1 or syntaxin molecules in cortical neurons (left panel, magenta). Fixed cells were co-immunostained with Alexa488-synapsin in order to label synapses and the images merged (middle panel, green). Scale bar: 5 µm. A merged image of left and middle panels corresponding to the boxed region is shown at a higher zoom (right panels). Scale bar 500 nm. Both syntaxin1a and munc18-1 single molecules are seen to cluster in synapses. b. The distribution of munc18 and syntaxin1a fluorescent fusion protein molecules is similar to the endogenous pattern. All frames containing either munc18-1 or syntaxin1a signals from single molecules were summed and displayed as an intensity image to approximate the diffraction-limited image (greyscale, top left panels). PALM localization maps show single molecules of PA-mCherry-syntaxin1a or PA-mCherry-munc18-1 clustering with eitherEGFP-munc18-1 or EGFP-syntaxin respectfully (bottom left panels). The boxed regions are displayed at a higher zoom (right panels). Scale bars: 500 nm.

Supplementary Figure 2

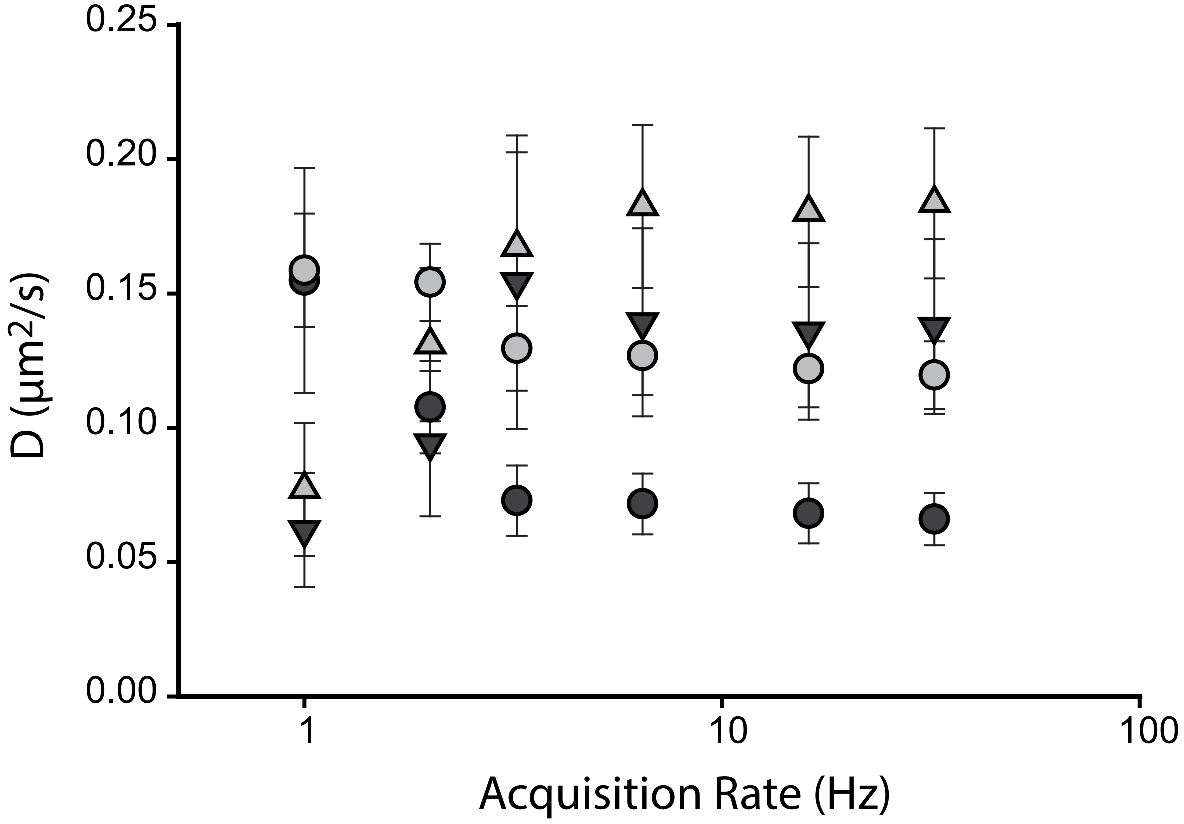

Supplementary Figure 2 – Effect of Sampling Rate on Apparent Diffusion Rate Extracted from FRAP Curves. The rate of acquisition used in FRAP experiments affects the fitted time constant of diffusion and therefore the apparent diffusion rate. When the same data are sampled at different rates (1 Hz, 2 Hz, 3.2 Hz, 6.5 Hz, 16.1 Hz and 32.3 Hz) there is clear variation in the reported diffusion rate (μm2/s) at acquisition rates of less than 3.2 Hz, after which the measurement plateaus. Exponential (light grey fill) models are used to fit data from labelled syntaxin1a (circles) and munc18-1 (triangles). n=7 at each point, and error bars represent the standard error in the mean. Of note, it is clear that syntaxin1a, an integral membrane protein, has a slower apparent diffusion rate that varies less with data acquisition rate than munc18-1. Munc18-1, a soluble protein that may or may not interact with syntaxin1a, on the other hand has a calculated diffusion rate that converges with that of syntaxin1a as the data acquisition rate increases, and this dependency plateaus at around 10 Hz. These data demonstrate that we cannot resolve the different behaviors of munc18-1 at sampling rates slower than the faster behavior revealed at around 10 Hz. These data support the FCS diffusion measurements (that are acquired on the microsecond timescale) as well as highlighting the danger of under-sampling cellular dynamics.

Supplementary Figure 3

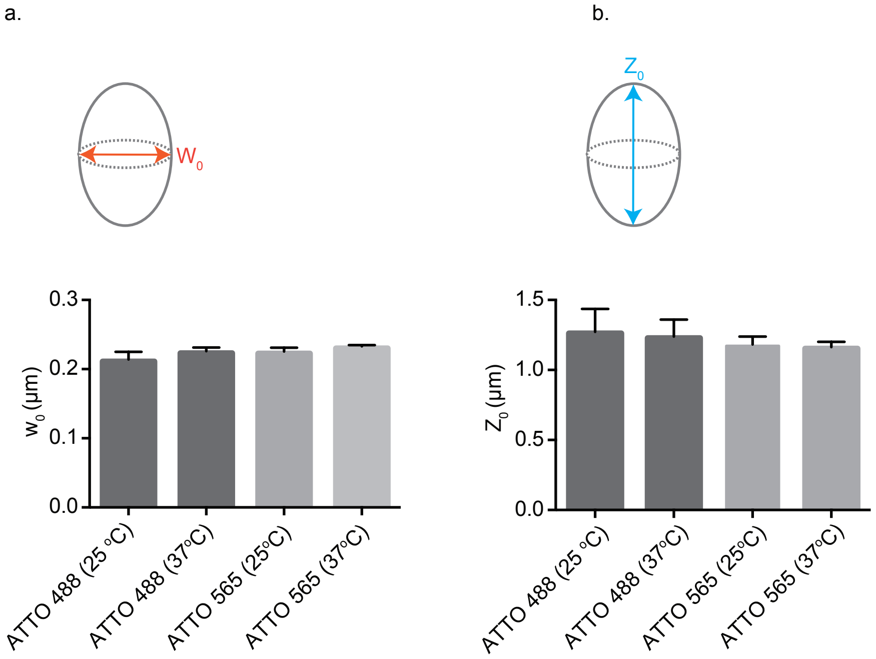

Supplementary Figure 3

Calibration of FCS effective volume. Prior to each FCS experiment the values for Kappa (the ratio of the axial and waist excitation spot dimensions) and Veff were determined using 10 nM Atto488 and Atto561 (Atto-Tec GmbH, Germany) standards in water at either 25oC or 37oC. These calibrations determined that the effective volume of the FCS spot was 0.29 ± 0.04 µm3 at 37oC; all in cellulo FCS measurements were made at this temperature. This volume is significantly smaller than the volume estimated recently using electron microscopy for central neuronal synaptosomes (0.37 ± 0.04 µm3; Ref 38). Bars show the calculated ‘waist’ (W) and axial ‘z’ (z) dimensions.

Supplementary Figure 4

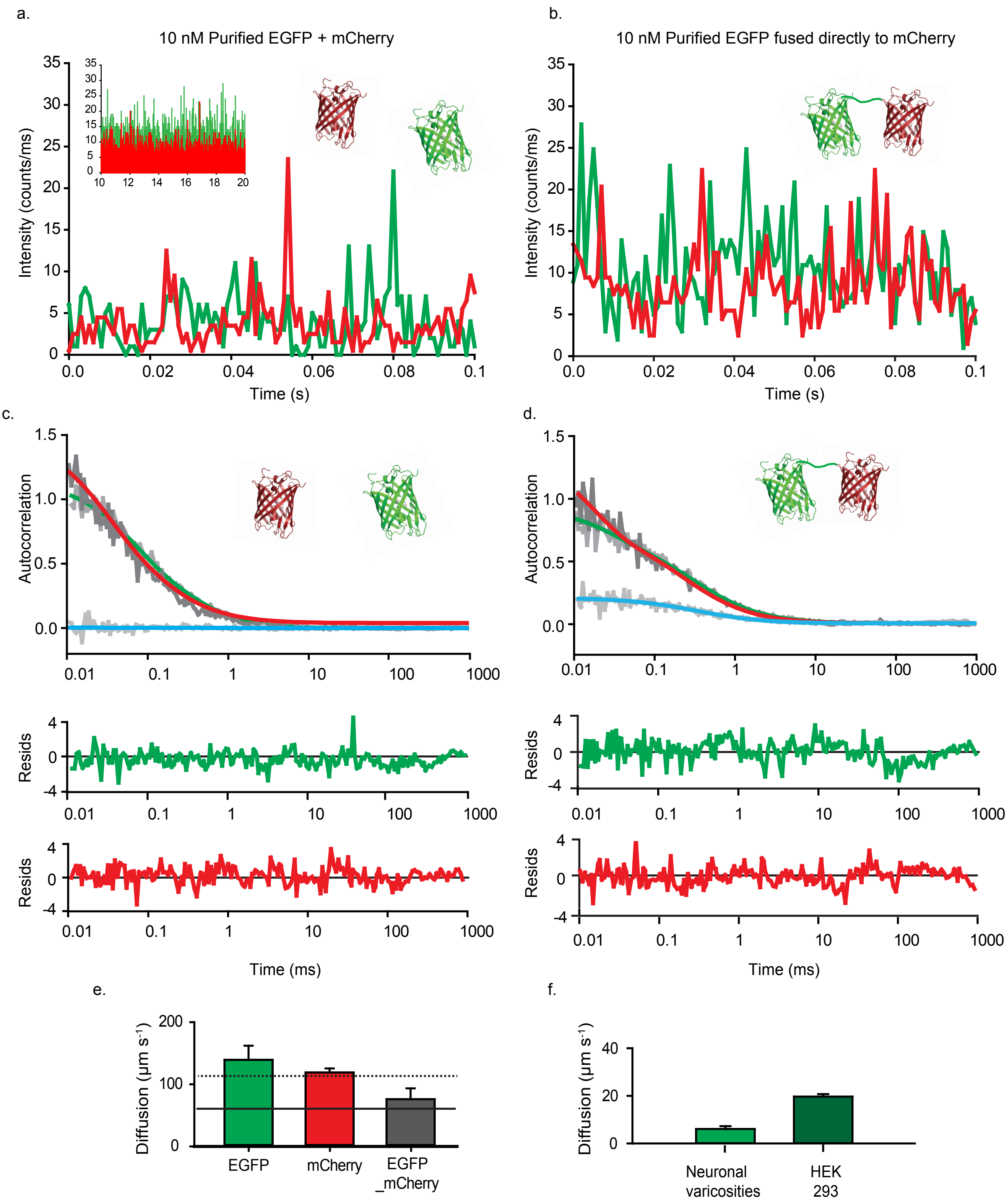

Supplementary Figure 4

FCS accurately reports diffusion rates in vitro and in vivo. a-b. Representative intensity (MCS) traces of purified EGFP, mCherry and of EGFP_mCherry fusion protein. c-d. Autocorrelation curves of the data presented in a and b. Fit residuals for the data (below). Cross correlation is shown as the blue fit line in both panels. Notice the increase in cross correlation for the EGFP mCherry fusion protein. e. Diffusion rates of purified fluorescent proteins at 37°C. Stokes Einstein estimated diffusion coefficients at 25°C (black line) and at 37°C (dashed black line). f. Measured diffusion rates of EGFP in neuronal varicosities and in HEK293 cells.

Supplementary Figure 5

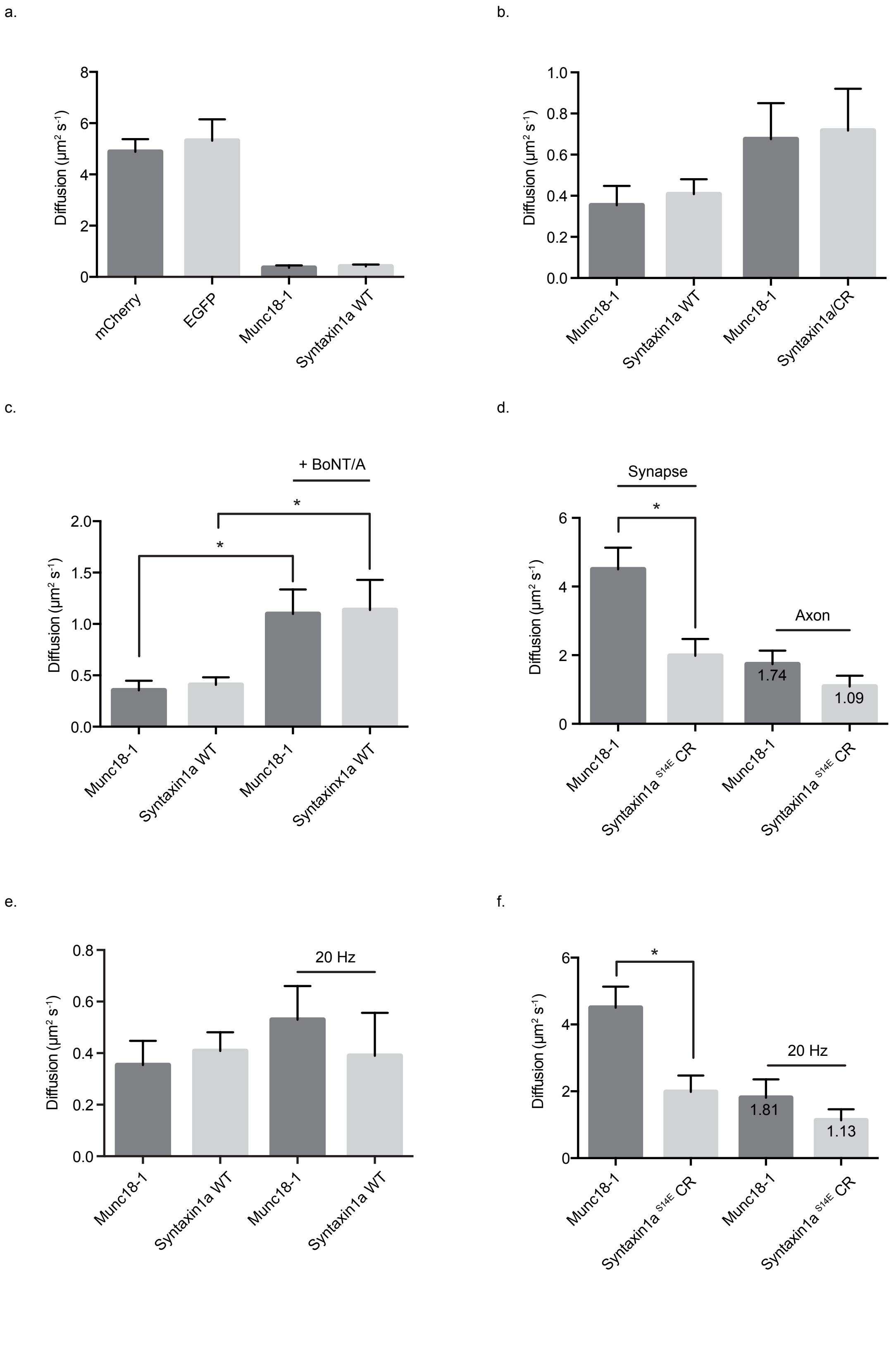

Supplementary Figure 5
Bar charts summarising all the FCS molecular diffusion data.

Supplementary Figure 6

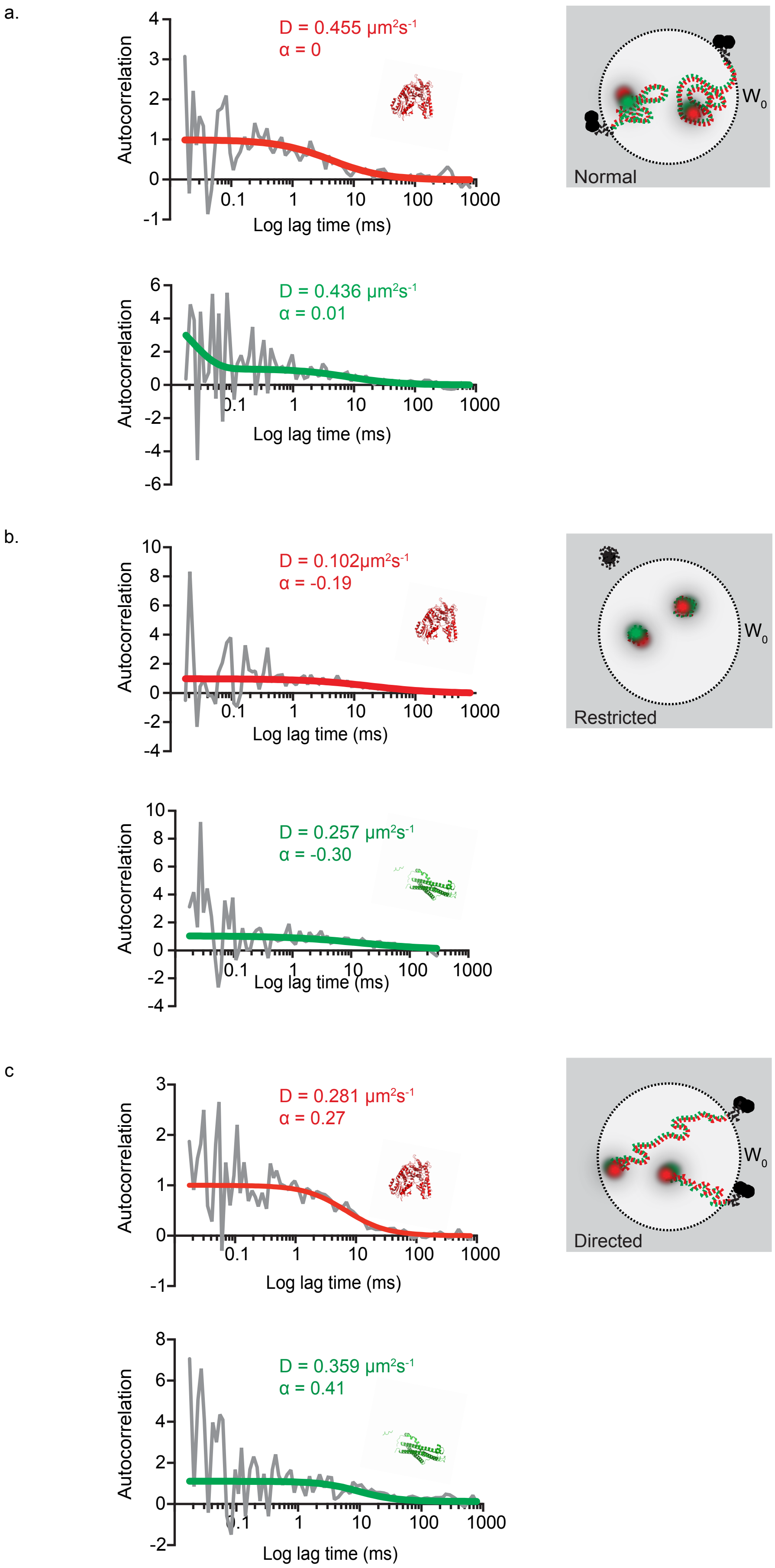

Supplementary Figure 6
Representative raw FCS autocorrelation and cross-correlation data with fitted curves. Raw data (grey lines) are overlaid with fitted autocorrelation functions (for munc18-1: red line, for syntaxin1a, green lines). The alpha value for each example fit is also shown. The cartoons display the molecular behaviors represented by each fitting model.

Supplementary Figure 7

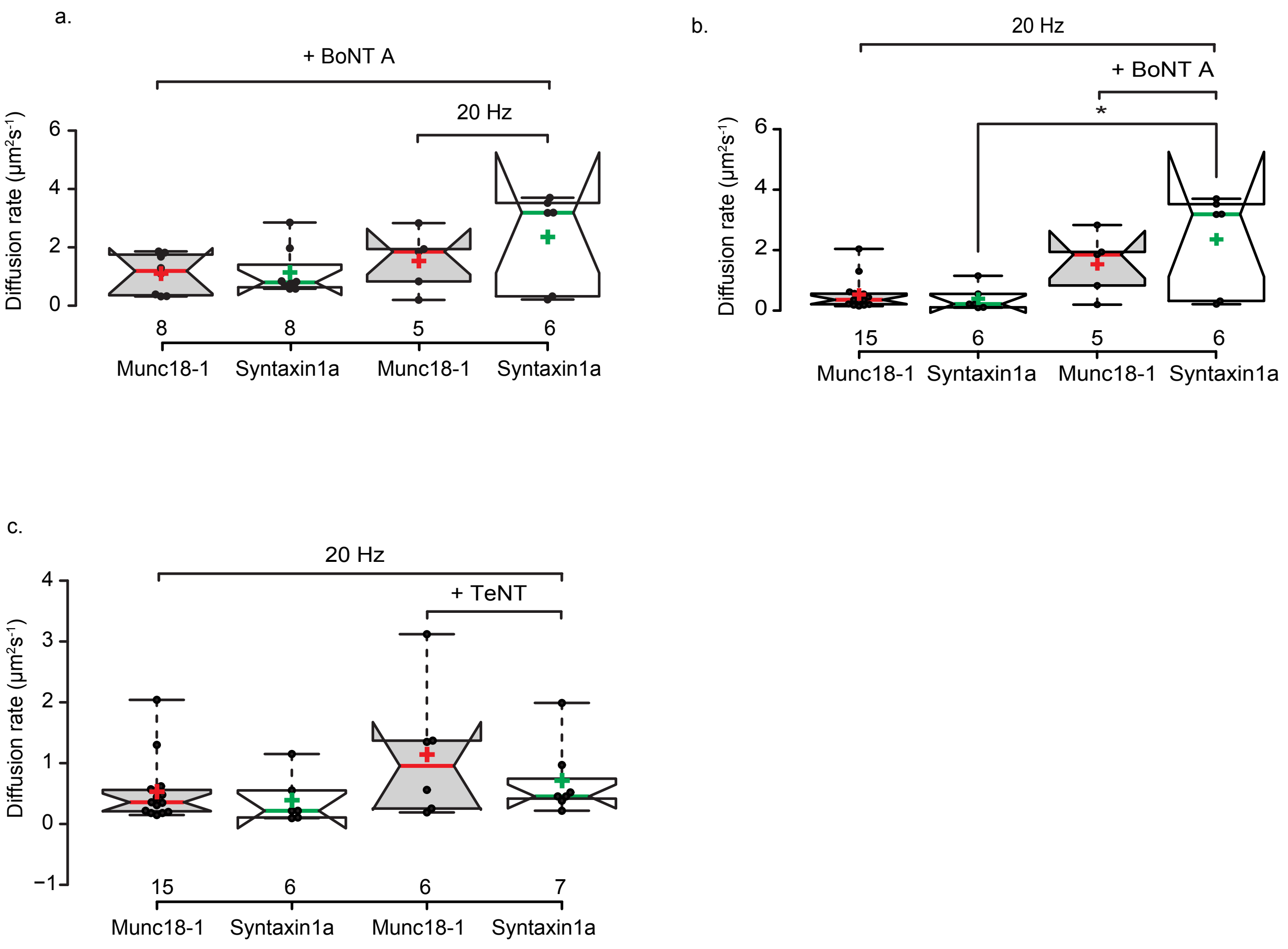

Supplementary Figure 7
BoNT/A cleaves proteolytically the C-terminal 9 amino acids from SNAP-25, halting the pathway to fusion at a late stage; this effect can be overcome by high Ca2+ concentrations, indicating that the exocytotic machinery is functional but arrested pre-fusion. These experiments may therefore reveal whether transient dissociations of syntaxin1a and munc18-1 occurred, on a timescale faster than we could detect in unhindered synapses. FCS studies as before (a) in BoNT/A-poisoned synapses found no effect on the syntaxin1a-munc18-1 interaction before or during depolarization (diffusion rates 1.68 ± 0.68 and 1.46 ± 0.66 µm2s-1, respectively, n = 9 measurements from 3 cells, mean ± SEM). b. Comparing these data with those acquired from untreated synapses showed that the syntaxin1a – munc18-1 complexes in BoNT/A-treated synapses increased in diffusion rate. C. Similar experiments using cells pre-treated with Tetanus toxin (TeNT) showed no effect on the rates of diffusion of the munc18-1 – syntaxin1a complex.

Supplementary Figure 8

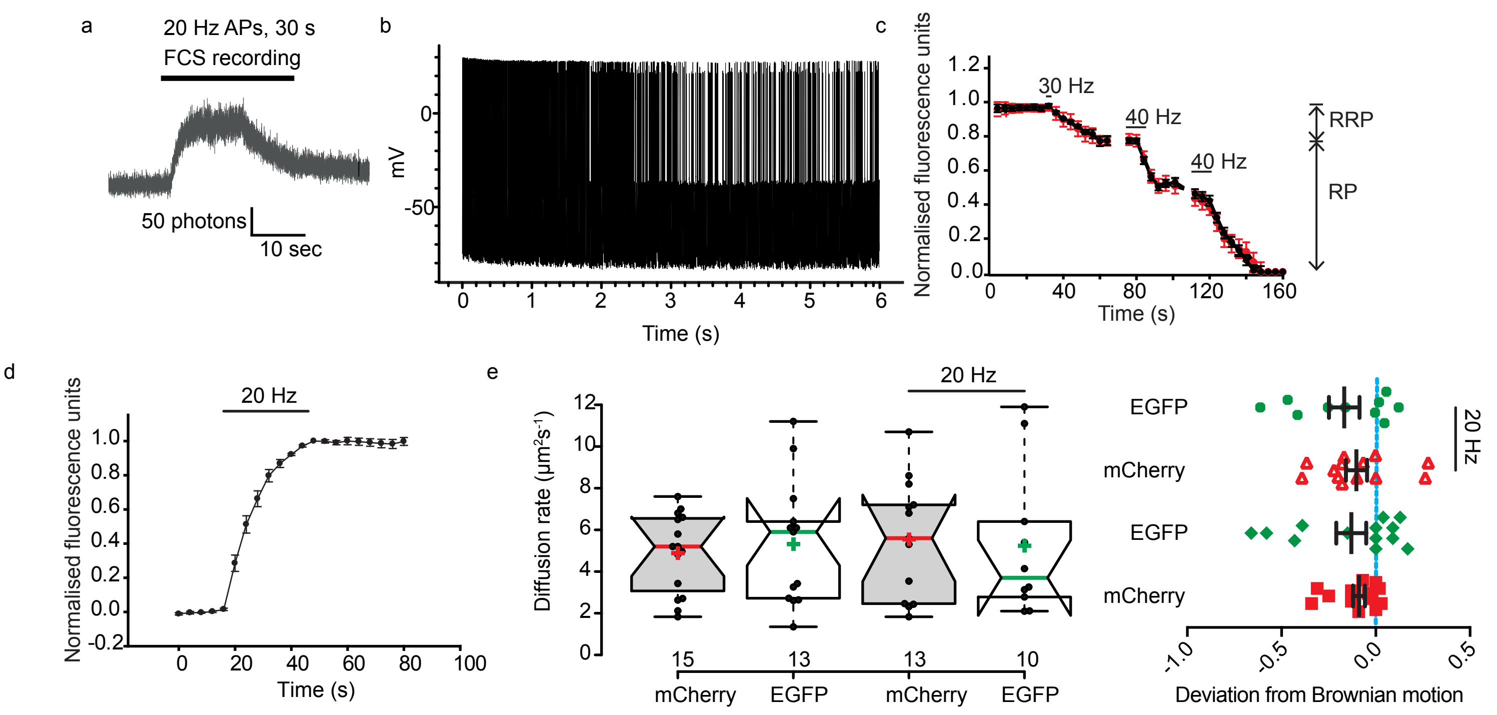

Supplementary Figure 8

Single molecule measurements in synapses during electrical depolarization. a. high-temporal-resolution Ca^{2+} dynamics measured from a single synapses during maximal stimulation (20 Hz action potentials, 30 s) show synaptic Ca2+ concentration reduces after ~20 s of depolarization. b. whole-cell current clamp of the same cells shows that action potentials are maintained c. FM-dye unloading experiments using untransfected (black symbols) and cells transfected with our fluorescent probes (red symbols) show no effect of transfection on the kinetics of exocytosis or vesicle pool size (RRP – readily releasable pool; RP – reserve pool). Bars and text indicate stimulation regime and times. d. SypHy fluorescence measurements during maximal stimulation (600 action potentials at 20 Hz) report that all of the recycling vesicle pool (RRP + RP) is released during the course of stimulation. e. Left panel - Box-plots of unfused mCherry and EGFP diffusion rates determined from FCS autocorrelations show no non-specific effect of maximal depolarization on these molecules in synapses. Right panel shows that unfused fluorescent proteins diffuse following a Brownian motion model.

Supplementary Figure 9

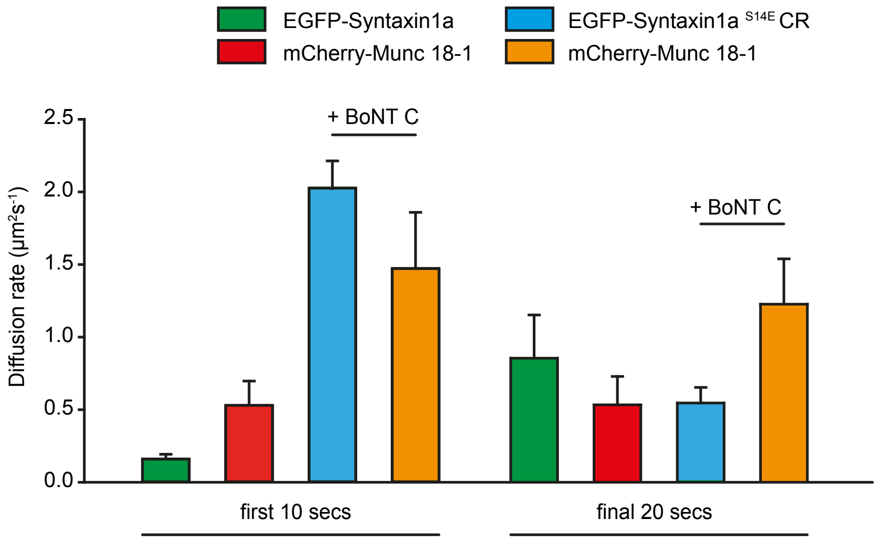

Supplementary Figure 9
Separating the FCS data into temporal blocks supports the FLIM – FRET analyses and reveals an interaction between syntaxin1aS14ECR and munc18-1 induced only after several seconds of depolarization.
